# Sexual Identity Development Milestones, Latent Profiles, and Proximal Minority Stressors in Australia’s Generation Z

**DOI:** 10.1177/09567976251372130

**Published:** 2025-09-10

**Authors:** William Warton, Michelle L. Byrne, Kelly-Ann Allen

**Affiliations:** 1 Psychological Sciences, Faculty of Medicine, Nursing and Health Sciences, Monash University; 2 Turner Institute for Brain and Mental Health, Faculty of Medicine, Nursing and Health Sciences, Monash University; 3 School of Educational Psychology and Counselling, Faculty of Education, Monash University

**Keywords:** sexual identity development, Generation Z, minority stress, milestones

## Abstract

This study examined the sequence and timing of sexual identity development (SID) milestones among Generation Z LGBTQ+ Australians, focusing on variations across subgroups and their relationship with minority stressors. The study included 490 Australian LGBTQ+ individuals aged 16 to 26, predominantly White (*n* = 389) and assigned female at birth (*n* = 402), with a balanced distribution between cisgender and gender-diverse participants. Demographic differences in the timing and achievement of SID milestones were found for sexual and gender identity. Latent profile analysis identified four distinct profiles, highlighting identity-centered and sex-centered sequences. Differences in rejection sensitivity, emotion dysregulation, and self-acceptance of sexual identity were noted across these profiles, but not for internalized homonegativity. Our findings indicate that SID trajectories do not strictly conform to discrete sexual or gender identity categories. The cross-sectional design limits causality inference, and findings are not generalizable to all LGBTQ+ young people or Generation Z more broadly.

Generation Z (individuals born between approximately 1997 and 2012) is navigating a cultural landscape marked by unprecedented representation of LGBQ+ people, as well as transgender and gender-nonconforming (TGNC) people, in media, popular culture, and education (Das & Farber, 2020; Riggs & McIntyre, 2022). Despite this, LGBTQ+ young people experience mental-health disparities and minority stressors comparable to, or worse than, those experienced by older cohorts (Frost et al., 2022; Meyer et al., 2021). At the same time, the age at which LGBTQ+ young people begin exploring their sexual identity has declined (Bishop et al., 2020; Hall et al., 2021; Layland et al., 2023). Sexual identity development (SID) now occurs alongside other adolescent stressors, such as puberty, bullying, and social belonging (Fish & Russell, 2022; Russell & Fish, 2019). However, most SID research focuses on older generations, leaving limited insights into Generation Z. This study explores SID milestone patterns and their links to minority stressors within Generation Z.

## Sexual Identity Development Milestones

LGBTQ+ people experience varied SID pathways in both the sequence and age of onset (Bishop et al., 2023; Hall et al., 2021). Typical sequences begin with attraction, followed by either sexual activity (sex-centered) or self-identifying as LGBTQ+ (identity-centered). Studies have shown that coming out, especially to family, typically marks the final milestone (Bishop et al., 2020, 2023; Meyer et al., 2021). The timing and pacing of SID milestones vary among LGBTQ+ subgroups. Cisgender male and monosexual individuals (e.g., lesbian or gay) have been reported to experience attraction, self-identity, and sexual activity earlier than cisgender females and bisexual individuals (Bishop et al., 2020; Hall et al., 2021; Martos et al., 2015). However, milestone dynamics for plurisexual identities (attracted to more than one gender/sex) are less understood (Galupo et al., 2017), although some evidence suggests pansexual and queer individuals reach milestones at ages similar to monosexuals and younger than bisexuals (Bishop et al., 2020). Evidence also suggests that racial-minority individuals often achieve relationship and disclosure milestones earlier than racial-majority individuals (Bishop et al., 2020; Mernitz et al., 2023), although findings vary depending on the milestone measurement across studies (Hall et al., 2021).

SID pathways among TGNC populations, and for individuals on the asexual spectrum, remain poorly understood because of limited research (Hall et al., 2021). Historically, studies on SID have predominantly focused on cisgender individuals, often overlooking the unique experiences of TGNC individuals. Yet TGNC individuals report distinct SID patterns, including variations in the age of first awareness and coming out (Layland et al., 2023). Meanwhile, asexual individuals may experience attraction and sexual activity differently, with the absence of sexual attraction marking a unique milestone not addressed by traditional models (McInroy et al., 2021). These gaps underscore the need for more inclusive research to fully understand SID experiences across LGBTQ+ identities.

## Milestones and Minority Stress

Minority stress, stemming from societal prejudice and stigma (Brooks, 1981; Meyer, 2003), has been linked to the health disparities experienced by LGBTQ+ populations (Dürrbaum & Sattler, 2020). Minority stress theory posits that LGBTQ+ health disparities are produced by socially embedded stressors related to stigma and prejudice, co-occurring with general life stressors (Meyer, 2003; Rivas-Koehl et al., 2023). This stress includes external (distal) sources, such as societal discrimination and violence, and internal (proximal) sources, through which stigma becomes internalized, leading to identity concealment, rejection sensitivity, and internalized homonegativity (internalized sexual stigma). Related to proximal stressors, emotion dysregulation serves as a psychological link between minority stress and mental health (Mann et al., 2022). Research indicates that faster progression through SID milestones, as seen in gay and bisexual men, correlates with increased discrimination, emotion dysregulation, and internalizing symptoms in adulthood (Rendina et al., 2019).

Research across cohorts suggests that, despite improved social contexts, distal stressors such as discrimination remain stable, highlighting the critical role of proximal stressors in LGBTQ+ mental health and suggesting that shifts in social attitudes may not significantly reduce minority stress (Meyer et al., 2021; Puckett et al., 2022). Milestones, as events anchored to specific developmental times, allow for unique sequences influenced by contextual factors. In older generations (millennials, Generation X), cross-sectional evidence links early adolescent SID milestones with greater internalizing symptoms in adulthood among cisgender monosexuals (Katz-Wise et al., 2017). Further research with cisgender male Generation Z adolescents shows delayed coming out when facing heightened internalized stigma (Moskowitz et al., 2022). A recent systematic review supports this, finding that lower internalized homonegativity facilitates identity disclosure (Son & Updegraff, 2023). These findings suggest that the timing and context of SID milestones significantly affect minority stress in LGBTQ+ individuals, reinforcing the need for studies exploring these dynamics within Generation Z, in which social acceptance and visibility might have complex impacts on mental health.

## Current Study

The study aimed to investigate the typical ages of SID milestones in Generation Z, differences across LGBTQ+ subgroups (sexual and gender identities), common sequences of milestones, and whether certain milestone sequences are associated with internalized homonegativity, rejection sensitivity, identity acceptance, and emotion regulation. An important strength of this study is its inclusion of TGNC individuals; prior SID research has largely focused on cisgender individuals exclusively (cf. Layland et al., 2023). Building on prior research, we expected milestone sequences to follow an identity-centered pattern, with self-identity preceding sexual activity (Bishop et al., 2023; Hall et al., 2021). Moreover, we expected to identify unique milestone sequences and pacing between monosexual participants and plurisexual participants (for a review, see Hall et al., 2021). Considering the emerging generational shift in milestone experiences (Bishop et al., 2020; Meyer et al., 2021), we hypothesized that milestones would be experienced earlier and be more condensed (i.e., faster pacing) for younger participants.

## Research Transparency Statement

### General disclosures

**Conflicts of interest:** Authors have no relevant financial or nonfinancial competing interests to report. All authors declare no conflicts of interest. **Funding:** This project was funded by the Turner Institute for Brain and Mental Health at Monash University. **Artificial intelligence:** No AI-assisted technologies were used in this research or the creation of this article. **Ethics:** The current study was approved by the Monash University Human Research Ethics Committee (ID 37035).

### Study disclosures

**Preregistration:** The aims, hypotheses, method, and analyses of this study were registered on July 25, 2023, during data collection but prior to any author viewing the data (https://osf.io/ynjsm). Data collection began on April 29, 2023. Minor deviations from the preregistration in the planned statistical analyses were made to maintain statistical rigor, as detailed in the Method section. **Materials:** All study materials are publicly available (https://doi.org/10.5281/zenodo.16552435). **Data:** All primary data are publicly available (https://doi.org/10.5281/zenodo.16552435). **Analysis scripts:** The analysis scripts used for the primary analyses are publicly available (https://doi.org/10.5281/zenodo.16552435). **Computational reproducibility:** The computational reproducibility of the results in the main article (but not in the Supplemental Material) has been independently confirmed by the journal’s STAR team.

## Method

### Sample and procedure

Participants who self-identified as heterosexual were excluded from the study. A total of 759 individuals took part in the survey. The survey included two attention-check questions one third and two thirds the way through the survey that asked participants to select a specific answer from a 5-point scale. Participants who failed at least one attention check (*n* = 85), had under a 95% completion rate (*n* = 171), or failed to meet recruitment criteria or did not consent to participate (*n* = 13) were excluded from the sample. Because of the high completion rate of the final sample, missing data were left in. The final analytic sample contained 490 Australian LGBTQ+ (all sexually and gender diverse) individuals aged between 16 and 26 years (*M* = 18.67 years, *SD* = 2.95) born during or after 1997. The sample was predominantly White (*n* = 389) and assigned female at birth (*n* = 402; assigned male at birth: *n* = 83). For a demographic breakdown of the analytic sample, see Table 1. For a summary of the chi-square analysis, refer to Section S1 and Table S1 in the Supplemental Material available online. Table S1 also contains the remaining demographic characteristics not used in the main analyses.

**Table 1.** Demographic Characteristics by Milestone Achievement

Demographic	Attraction	Self-identity	S-S SA	O-S SA	S-S Rel	O-S Rel	First disclosure	Familial disclosure	*N*
SAB									
Male	76 (15.5)	82 (16.7)	46 (9.4)	30 (6.1)	39 (8.0)	33 (6.7)	81 (16.5)	57 (11.6)	83
Female	355 (72.4)	400 (81.6)	203 (41.4)	151 (30.8)	209 (42.7)	192 (39.2)	396 (80.8)	314 (64.1)	402
χ^2^ (*df*), *p*	0.79 (1), = .37	0.00 (1), > .99	0.61 (1), = .43	0.01 (1), = .90	0.50 (1), = .48	1.51 (1), = .22	0.58 (1), = .45	2.90 (1), = .09	
Intersex	1 (0.2)	2 (0.4)	1 (0.2)	—	1 (0.2)	2 (0.4)	2 (0.4)	2 (0.4)	2
Prefer not to say	3 (0.6)	3 (0.6)	1 (0.2)	1 (0.2)	2 (0.4)	3 (0.6)	3 (0.6)	3 (0.6)	3
Gender identity									
Cisgender male	46 (9.4)	49 (10.0)	27 (5.5)	15 (3.1)	20 (4.1)	16 (3.3)	48 (9.8)	31 (6.3)	50
Cisgender female	165 (33.7)	180 (36.7)	85 (17.3)	69 (14.1)	76 (15.5)	84 (17.1)	179 (36.5)	126 (25.7)	182
Gender expansive	137 (28.0)	162 (33.1)	85 (17.3)	60 (12.2)	93 (19.0)	82 (16.7)	160 (32.7)	136 (27.8)	162
Transfeminine	13 (2.7)	15 (3.1)	8 (1.6)	7 (1.4)	8 (1.6)	7 (1.4)	15 (3.1)	11 (2.2)	15
Transmasculine	62 (12.7)	68 (13.9)	39 (8.0)	28 (5.7)	48 (9.8)	36 (7.3)	67 (13.7)	63 (12.9)	68
χ^2^ (*df*), *p*	4.12 (4), = .39	3.70 (4), = .45	2.79 (4), = .59	2.65 (4), = .62	21.76 (4), < .001	6.51 (4), = .16	7.35 (4), = .12	26.44 (4), < .001	
Uncategorized^a^	12 (2.4)	13 (2.7)	7 (1.4)	3 (0.6)	6 (1.2)	5 (1.0)	13 (2.7)	9 (1.8)	13
Sexual identity									
Monosexual	164 (33.5)	167 (34.1)	103 (21.0)	46 (9.4)	102 (20.8)	56 (11.4)	165 (33.7)	133 (27.1)	167
Plurisexual	202 (41.2)	211 (43.1)	112 (22.9)	108 (22.0)	100 (20.4)	127 (25.9)	210 (42.9)	161 (32.9)	212
Asexual spectrum	39 (8.0)	80 (16.3)	16 (3.3)	14 (2.9)	30 (6.1)	30 (6.1)	79 (16.1)	61 (12.4)	80
Queer-plus	30 (6.1)	29 (5.9)	20 (4.1)	14 (2.9)	19 (3.9)	17 (3.5)	28 (5.7)	21 (4.3)	31
χ^2^ (*df*), *p*	166.29 (3), < .001	18.96 (3), < .001	40.52 (3), < .001	37.13 (3), < .001	15.17 (3), = .002^b^	30.55 (3), < .001	10.89 (3), = .012^b^	2.28 (3), = .52	
Race/ethnicity^c^									
White	347 (70.8)	388 (79.2)	204 (41.6)	145 (29.6)	202 (41.2)	181 (36.9)	382 (78.0)	311 (63.5)	389
POC	82 (16.7)	93 (19.0)	46 (9.4)	35 (7.1)	48 (9.8)	47 (9.6)	94 (19.2)	63 (12.9)	94
χ^2^ (*df*), *p*	0.33 (1), = .57	0.04 (1), = .84	0.29 (1), = .59	0.00 (1), > .99	0.00 (1), = .97	0.22 (1), = .64	0.13 (1), = .72	6.52 (1), = .011^b^	
Prefer not to say^d^	6 (1.2)	6 (1.2)	1 (0.2)	2 (0.4)	1 (0.2)	2 (0.4)	6 (1.2)	2 (0.4)	7
Education									
In high school	231 (47.1)	261 (53.3)	103 (21.0)	66 (13.5)	127 (25.9)	110 (22.4)	260 (53.1)	201 (41.0)	264
Not in high school	201 (41.0)	222 (45.3)	148 (30.2)	116 (23.7)	123 (25.1)	118 (24.1)	218 (44.5)	173 (35.3)	222
χ^2^ (*df*), *p*	0.31 (1), = .57	1.02 (1), = .31	37.26 (1), < .001	37.96 (1), < .001	2.29 (1), = .13	6.18 (1), = .013^b^	0.00 (1), > .99	0.13 (1), = .72	
Prefer not to say	3 (0.6)	4 (0.8)	—	—	1 (0.2)	2 (0.4)	4 (0.8)	2 (0.4)	4
Total achieved	435 (88.8)	487^e^ (99.4)	251 (51.2)	182 (37.1)	251 (51.2)	230 (46.9)	482 (98.4)	376 (76.7)	

Note: All values are *n* (%) unless otherwise indicated. Chi-square analysis results (including Bonferroni-corrected *p* values) indicate significant associations between demographics and milestone achievement. For a summary of the chi-square analysis, refer to Section S1 and Table S1 in the Supplemental Material available online. Table S1 also contains the remaining demographic characteristics not used in the main analyses. S-S SA = same-sex sexual activity; O-S SA = opposite-sex sexual activity; S-S Rel = same-sex relationship; O-S Rel = opposite-sex relationship; SAB = sex assigned at birth; POC = person of color. ^a^Uncategorized gender identities include those who were intersex at birth and identified their gender as intersex (*n* = 1), did not disclose a gender identity (*n* = 6) or sex assigned at birth (*n* = 2), or had a lengthy text entry (*n* = 4). These were not included in the chi-square analyses. ^b^This value is not significant after adjusting for multiple comparisons. ^c^Asian, mixed-race, Black, Indian, and Pasifika/Maori identities and unique text entries were combined as POC because of a low cell count for these groups. A full demographic breakdown of race/ethnicity can be provided on request. ^d^Responses that were missing (*n* = 4) and participants who preferred not to say (*n* = 3) were combined. ^e^Three participants who identified as questioning also did not achieve the self-identity milestone.

This study used multiple response options and text entries to capture the diversity of participants’ sexual and gender identities, aligning with the coalition approach described by Klysing et al. (2024) to balance understanding both shared and unique aspects of LGBTQ+ experiences. This approach enabled us to address the collective challenges of LGBTQ+ young people in navigating SID while highlighting areas in which future subgroup-specific research can further expand our understanding of SID. It is critical to recognize that sexual identity, gender identity, and sex characteristics are distinct but interconnected facets of identity. The interplay of these dimensions can result in diverse developmental trajectories (Hereth et al., 2020). Sexual orientation refers to an individual’s patterns of emotional, romantic, or sexual attraction to others, which may include same-sex, opposite-sex, or multiple-sex attractions. Gender identity refers to someone’s internal sense of their gender, or lack thereof, which may be inconsistent with their sex assigned at birth. In contrast, sex characteristics encompass biological attributes such as chromosomes, hormones, and reproductive or anatomical traits, which can include variations commonly associated with intersex individuals.

Participants from the analytic sample were categorized into five mutually exclusive gender groupings regardless of sexual identity, consistent with prior research (Flentje et al., 2022): cisgender sexually diverse men (sex assigned at birth and gender identity were both male), cisgender sexually diverse women (sex assigned at birth and gender identity were both female), gender-expansive individuals (selected a gender identity or multiple that were not reflective of a gender binary and did not match sex assigned at birth), transfeminine individuals (reported identity as transgender and/or female with sex assigned at birth being male), and transmasculine individuals (reported identity as transgender and/or male with sex assigned at birth being female). Participants were also categorized into four mutually exclusive sexual identity groupings: monosexual (attracted to only one gender/sex), plurisexual (attracted to more than one gender/sex), asexual spectrum, and queer-plus (nonspecific to first three options but do not identify as heterosexual).

To avoid restrictive labeling, especially for the current sample of young people who may not be ready to describe their sexual orientation but may be able to define their sexual attraction, and on the basis of recommendations by Deardorff et al. (2019), we included options such as “I am mostly attracted to girls” and “I am mostly attracted to boys” when measuring sexual orientation. The increase in the adoption of newer diverse identity labels, such as pansexual and queer, has been coupled with a reconceptualization of bisexuality (Galupo et al., 2014, 2017). Research has found that, compared with monosexual individuals, plurisexual individuals are less likely to feel that their sexual identity fully represents their sexuality and are more inclined to provide additional context about their identity labels (Dyar et al., 2015; Galupo et al., 2015). The fundamental notion of bisexuality begins to transform when considering gender/sex and when attempting to deconstruct heteronormative binaries (Cipriano et al., 2022). Because we were focused on the achievement of SID milestones, it seemed prudent to group bisexual and pansexual participants together as plurisexual individuals because their experience of sexual interactions and relationships during identity development is fundamentally different from monosexual individuals (Galupo et al., 2017). This approach has similarly been adopted by recent research with diverse sexual identity and gender identity samples (Feinstein et al., 2023).

Although we included participants who identified as intersex, we acknowledge that not all individuals who are intersex may be aware of their status at birth. Some intersex individuals discover their intersex traits only later in life, often during medical interventions or health evaluations, which may impact their understanding of identity and their experiences of milestones related to sexual identity and gender identity (Hart & Shakespeare-Finch, 2022). Our demographic measures allowed participants to self-identify as intersex or describe their sex characteristics in an open-ended format. However, those unaware of their intersex status at the time may not have self-identified, limiting the capture of diverse intersex experiences.

#### Procedure

The current study was approved by the Monash University Human Research Ethics Committee (ID 37035). Data were collected through a novel survey hosted by Qualtrics from May 2023 to September 2023. Participants were recruited through paid social media advertising on Facebook and Instagram. Because this study was exploratory, no a priori power analyses or sample size estimations were made. The recruitment timeline was determined purely by advertising budget, with the goal to recruit as large a sample as possible within this constraint. Informed consent was obtained through a detailed landing page containing the explanatory statement for the project. If participants elected to enter into the prize draw at the end of the survey to receive one of five $50 electronic gift cards, they were taken to a secondary survey that detached their email from their original survey responses to maintain anonymity.

### Measures

#### Demographics

Demographic information included age, current level of education, race/ethnicity, religious identity, disability status, neurodivergence, sexual identity, gender identity, and sex assigned at birth (for a summary and results of those demographic characteristics not included in the main analyses, see Section S2 in the Supplemental Material). The state of residence within Australia was also collected to confirm eligibility. Participants could select multiple response options for race/ethnicity, sexual identity, and gender identity. Because of low responses for groups, race/ethnicity was simplified into two categories for analysis: White (for those who only chose “White”) and person of color (for anyone who selected a different race, more than one race, or indicated their race was not included on the list). For sexual identity, participants were asked, “What do you sexually identify as? Please select all that apply.” Response options included lesbian; mostly attracted to girls; gay; mostly attracted to boys; straight (heterosexual); bisexual; queer; questioning; asexual; pansexual; preferred sexual identity (or at least one of them) not listed (participants could elaborate in an open text field); and prefer not to say. Participants were also asked to identify their gender. Options included male/man; female/woman; nonbinary; gender fluid; trans/gender diverse; preferred gender identity (or at least one of them) not listed (again with the option of elaborating in an open text field); and prefer not to say.

#### Sexual identity development milestones

The SID milestones were measured with 11 questions that assessed the age at which participants first achieved the following milestones: attraction, self-identity, same-sex sexual activity (with the same gender/sex or someone who is nonbinary or gender fluid), opposite-sex sexual activity, same-sex romantic relationship (with the same gender/sex or someone who is nonbinary or gender fluid), opposite-sex romantic relationship, disclosure (to anyone), and coming out to an immediate family member. Each milestone was measured using a question that directly asked the age at which the event occurred; for example, “How old were you when you first consensually engaged in sexual activity with someone of the same sex as you, or someone who is nonbinary or gender fluid?” There were two data types for each milestone: binary data containing information on whether the milestone occurred or did not occur and continuous data containing the specific age in years for when the milestone occurred. Participants had the option to select “I have not experienced this yet,” “This is not relevant to me,” “This is not applicable to me,” or “I have not done this yet” where appropriate for each milestone.

#### Minority stress variables

The constructs chosen for this study—internalized homonegativity, rejection sensitivity, identity acceptance, and emotion dysregulation—are well aligned with minority stress theory because they each represent key dimensions of proximal stress. Internalized homonegativity and rejection sensitivity, for example, capture the ways in which negative societal messages are internalized, leading to self-stigma and heightened vigilance against perceived rejection. Identity acceptance was included to examine the counterbalance to these stressors because self-acceptance can mitigate internalized stigma and promote resilience in LGBTQ+ individuals (Son & Updegraff, 2023).

##### Rejection related to sexual orientation

Expectations of rejection related to sexual orientation were measured using a revised version of the 14-item Sexual Minority Adolescent Rejection Sensitivity Scale (Kiekens et al., 2023; revised with permission). In the context of the scale, “sexual minority” refers to individuals whose sexual orientation deviates from heterosexual norms, including lesbian, gay, bisexual, queer, and other nonheterosexual identities. The rejection sensitivity construct reflects the extent to which individuals anticipate and react to potential rejection specifically tied to their sexual identity, encompassing both anxiety about rejection and expectations of it occurring in ambiguous social situations. All 14 items were measured on two 6-point Likert scales. An example item is “Imagine that you are instructed to work on an assignment/task with a partner and no one wants to work with you.” The first question measured anxiety and asked “How anxious/concerned would you be that [situation described in scenario] occurred because of your sexual orientation?” (1 = *very unconcerned*, 6 = *very concerned*). The second question measured expected rejection and asked “How likely is it that [situation described in scenario] occurred because of your sexual orientation?” (1 = *very unlikely*, 6 = *very likely*). The revisions involved altering the wording of the scenarios to include school/university/work to maintain relevance for the Generation Z sample. In the current study sample, scale reliability (α) was .89.

##### Internalized homonegativity

The internalized homonegativity subscale of the Lesbian, Gay, and Bisexual Identity Scale (Mohr & Kendra, 2011) was used to measure internalized homophobia/homonegativity. The construct of internalized homonegativity reflects internalized stigma toward nonheterosexual identities, including monosexual and plurisexual identities. Although the term “homonegativity” is used to align with established research and validated measures, we note that it encompasses negative internalized perceptions related to a variety of nonheterosexual identities. This construct captures the degree to which individuals internalize negative societal beliefs or prejudices about diverse sexual identities, manifesting in self-directed stigma or discomfort with their own sexual identity. The three items were rated on a 6-point scale from 1 (*disagree strongly*) to 6 (*agree strongly*). An example item is “If it were possible, I would choose to be straight.” It has recently been recommended that this subscale be used because it does not conflate internalized homonegativity with distress like other common measures of internalized homonegativity do (Lefevor et al., 2023). In this sample, scale reliability (α) was .88.

##### Emotion dysregulation

The brief 16-item Difficulties in Emotion Regulation Scale (DERS-16; Bjureberg et al., 2016) was used to measure emotion dysregulation. This construct measures the difficulty individuals experience in understanding, regulating, or responding to their emotional states. The 16-item version has retained high internal consistency (α = .92) compared with the original 36-item version (Gratz & Roemer, 2004), including in both community and clinical samples (Charak et al., 2019; Miguel et al., 2017). Items were measured on a 5-point scale from 1 (*almost never*) to 5 (*almost always*). An example item is “I have difficulty making sense out of my feelings.” Total scores on the DERS-16 range from 16 to 80, where higher scores indicate greater levels of emotion dysregulation. In this sample, full scale reliability (α) was .84.

##### Self-acceptance of sexual identity

To measure self-acceptance of sexual identity, the 10-item Self-Acceptance of Sexuality Inventory was used (Camp et al., 2022). This construct reflects the degree to which individuals accept and feel comfortable with their sexual identity as an integral part of their self-concept. It encompasses a neutral to positive stance characterized by the absence of internal conflict about one’s sexuality, as well as the integration of this aspect of identity without judgment or rejection. For example, a high level of self-acceptance may be indicated by responses to items such as “I accept my sexuality” and an absence of agreement with statements such as “I feel in conflict about my sexuality.” The items were measured on a scale from 1 (*totally true for me*) to 5 (*totally untrue for me*). Full scale reliability (α) was .89.

#### Covariates

Milestone timing differs from current age. Variables other than milestones were measured at present, so the time between milestones and proximal minority stress measurements varied with participant age. Therefore, age at survey completion was included as a covariate because younger participants may have recently experienced milestones that affected their minority stress experiences differently than older participants. Retrospective perceived pubertal timing was also included as a covariate because early or late pubertal development may impact the age at which milestones are first experienced. Although our preregistration initially specified multiple items from the Pubertal Development Scale (Zehr et al., 2007), only one item was ultimately used because of low internal consistency across the full scale (α = .58). The chosen item “In general, do you think your development was any earlier or later than most other people your age?” broadly captures participants’ perceived timing relative to peers. The item was measured on a scale from 1 to 5 (1 = *much earlier*, 3 = *about the same*, 5 = *much later*).

### Analytic plan

Data were managed and analyzed using R Version 4.3.3 in RStudio Desktop Version 2023.12.1 and with Mplus 8 (Muthén & Muthén, 2017; R Core Team, 2024; RStudio Team, 2020). The analytic plan comprised several steps to address the aims of the current study. First, chi-square analyses with a Bonferroni correction were conducted to examine demographic differences between participants who did and did not report milestone achievement. Next, a series of analyses of covariance (ANCOVAs) were performed to test demographic differences in individual milestone timing. Participant age and perceived pubertal timing were included as covariates. To further explore these findings, post hoc analyses using Tukey’s honestly significant difference test were conducted to test significant ANCOVAs. Demographic characteristics explored in the main analyses included sex assigned at birth, sexual identity, gender identity, school status, and race/ethnicity. For a summary of the other characteristics, refer to Section S2.

Last, a latent profile analysis (LPA) was conducted to categorize individuals on the basis of distinct patterns of SID milestone achievement. Milestones included were attraction, self-identity, same-sex sexual activity, opposite-sex sexual activity, same-sex romantic relationship, opposite-sex romantic relationship, disclosure (to anyone), and coming out to an immediate family member. Considering the variability of identity development, it was expected that some milestones might not yet be achieved, influencing profile formation. To account for this, each milestone was coded with a binary indicator, where the experience of a milestone was coded 1 and the absence of that experience 0. This binary coding allowed the LPA to incorporate both achieved and unachieved milestones, capturing developmental variability within this young cohort. A regression specification was used to model the likelihood of each milestone’s achievement status within each profile. This approach enabled the identification of distinct milestone patterns across profiles while accommodating the potential for the future achievement of milestones not yet completed by some participants.

The default LPA model in Mplus freely estimates the mean age for the milestones in each profile while assuming equal variances and covariances across profiles. The variance represents the variability around the mean age of milestone achievement in each profile, whereas the covariance between milestones represents the timing between milestones. Therefore, constraining these would mask the potential differences in variability across profiles in the sequence and timing of the milestones. Bishop et al. (2023) specified an LPA model with means and variances freely estimated, which is the most theoretically meaningful. The binary indicators were regressed on the corresponding age of achievement milestone so that the likelihood of achieving a milestone would be modeled to depend directly on the age at which the milestone occurs. This captures how early or late achievement of a milestone influences whether it is achieved at the point of data collection. The age milestone data were also censored above in Mplus, which is essentially right censoring, indicating that the event (milestone achievement) might not occur by the end of the study but could still have happened later.

To account for varied experiences of milestone achievement, weighted values were calculated within each profile. This approach reflects the estimated share of participants within a given profile who did not report experiencing each milestone, adjusting for the individual’s likelihood of profile membership. To facilitate this, milestone indicators were reverse coded so that milestones not experienced were assigned a value of 1 and experienced milestones a value of 0. The binary indicator was then multiplied by the posterior probability of profile membership for each participant, generating individual-level weighted scores. These scores were then aggregated across participants for each profile and milestone to get the overall weighted proportion, which was converted to a percentage for reporting in tables and figures.

Models up to a five-profile solution were estimated, beyond which models did not converge. Each model utilized maximum likelihood estimation with robust standard errors and Monte Carlo integration, set at 500 points for accuracy and computational efficiency. The final model was selected on the basis of theoretical considerations, relative entropy for profile distinction, and proportions of model-estimated and most likely profile assignments (Bauer, 2022). For more details on profile selection, refer to Section S3. Profile membership probabilities were then exported into R, where participants were assigned profile memberships. The Bolck, Croon, and Hagenaars three-step method was used to assess minority stress variables as outcomes of profile membership while controlling for age and pubertal timing (Bakk & Kuha, 2021; Bolck et al., 2004). This method performs well when assessing the means of continuous distal outcomes across profiles in an LPA and does not assume equality of variances across profiles. The method directly adjusts for classification errors by incorporating the uncertainty in profile membership assignments (Bakk & Kuha, 2021).

#### Exploratory analyses

Several exploratory analyses not included in the original preregistration were conducted to enhance understanding of SID patterns and test the robustness of the findings. Specifically, sensitivity analyses were performed separating participants identifying solely as bisexual from the plurisexual group to investigate whether any observed differences between plurisexual and monosexual groups were influenced by this subgroup. Additionally, a sensitivity analysis was conducted by excluding participants who identified as being on the asexual spectrum, as well as one excluding the attraction milestone entirely, to examine how the presence of asexual participants might impact milestone sequencing and profile composition within the LPA. These exploratory analyses allowed for an assessment of the stability of the profile structures and further validation of the distinct SID patterns identified.

## Results

### Differences in age of milestone achievement

The significant post hoc tests from the ANCOVAs testing demographic differences in the age at which each milestone was first achieved are presented in Table 2. Although some of the ANCOVAs indicated overall significant differences among groups, not all pairwise comparisons in the post hoc tests reached statistical significance.

**Table 2.** ANCOVA Post Hoc Pairwise Comparisons of Demographic Differences in Age of Milestone Achievement

Milestone	Group 1	Group 2	Age Difference in Achievement	*p*
Attraction	—	—	—	—
Self-identity	Assigned female at birth	Assigned male at birth	Assigned female younger	.02
	Cisgender female	Gender expansive	Gender expansive younger	< .001
	Cisgender female	Transmasculine	Transmasculine younger	< .001
	Asexual spectrum	Monosexual	Monosexual younger	.005
Same-sex sexual activity	Queer-plus	Monosexual	Queer-plus younger	.02
Opposite-sex sexual activity	—	—	—	—
Same-sex relationship	Plurisexual	Monosexual	Plurisexual younger	.03
	Queer-plus	Monosexual	Queer-plus younger	.02
	White	POC	POC younger	.008
Opposite-sex relationship	—	—	—	—
First disclosure	Cisgender female	Gender expansive	Gender expansive younger	.01
	Cisgender female	Transmasculine	Transmasculine younger	< .001
	Monosexual	Asexual spectrum	Monosexual younger	.04
Familial disclosure	Transmasculine	Cisgender female	Transmasculine younger	.02
	Transmasculine	Transfeminine	Transmasculine younger	.047

Note: POC = person of color.

#### Attraction

The results indicated significant group differences in age at attraction for sex assigned at birth, *F*(1, 322) = 4.31, *p* = .04, school status, *F*(1, 322) = 35.37, *p* < .001, and gender identity, *F*(4, 322) = 4.11, *p* = .003. However, no significant effects were found for sexual identity, *F*(3, 322) = 0.11, *p* = .95, and race/ethnicity, *F*(1, 322) = 0.31, *p* = .58. Post hoc tests revealed no significant differences between these groups.

#### Self-identity

The results indicated significant group differences in age at self-identification for sex assigned at birth, *F*(1, 357) = 8.59, *p* = .004, school status, *F*(1, 357) = 87.44, *p* < .001, gender identity, *F*(4, 357) = 12.01, *p* < .001, and sexual identity, *F*(3, 357) = 3.76, *p* = .01. Conversely, no significant effects were found for race/ethnicity, *F*(1, 357) = 0.45, *p* = .50. Self-identity was achieved earlier for participants assigned female at birth (*M* = 13.36 years, *SE* = 0.35) than those assigned male at birth (*M* = 14.91 years, *SE* = 0.47, *p* = .02). Self-identity was achieved later for cisgender female participants (*M* = 15.30 years, *SE =* 0.43) than gender-expansive participants (*M* = 14.19 years, *SE =* 0.38, *p* < .001) or transmasculine participants (*M* = 13.86 years, *SE* = 0.47, *p* < .001). Moreover, participants who identified as being on the asexual spectrum achieved self-identity later (*M* = 14.80 years, *SE* = 0.34) than participants who identified as monosexual (*M* = 13.66 years, *SE =* 0.26, *p* = .005).

#### Same-sex sexual activity

The results indicated no significant group differences in age at same-sex sexual activity for sex assigned at birth, *F*(1, 182) = 2.50, *p* = .12, and gender identity, *F*(4, 182) = 1.77, *p* = .14. Significant effects were observed for school status, *F*(1, 182) = 30.45, *p* < .001, and sexual identity, *F*(3, 182) = 3.51, *p* = .02. Race/ethnicity, *F*(1, 182) = 1.15, *p* = .28, was not significant. Post hoc tests indicated a significant difference in age of same-sex sexual activity for participants who identified as queer-plus (*M* = 14.57 years, *SE* = 0.72) and those who identified as monosexual (*M* = 16.47 years, *SE* = 0.44, *p* = .02).

#### Opposite-sex sexual activity

The results indicated no significant group differences in age at opposite-sex sexual activity for sex assigned at birth, *F*(1, 129) = 0.82, *p* = .37. Similarly, no significant effects were found for gender identity, *F*(4, 129) = 1.82, *p* = .13, sexual identity, *F*(3, 129) = 0.46, *p* = .71, and race/ethnicity, *F*(1, 129) = 0.04, *p* = .85. School status, *F*(1, 129) = 30.79, *p* < .001, was found to be significant. However, post hoc tests revealed no significant differences between groups.

#### Same-sex relationship

The results indicated significant group differences in age at same-sex relationship for sex assigned at birth, *F*(1, 181) = 23.59, *p* < .001, and school status, *F*(1, 181) = 81.70, *p* < .001. Significant effects were also found for gender identity, *F*(4, 181) = 3.68, *p* = .007, sexual identity, *F*(3, 181) = 3.41, *p* = .02, and race/ethnicity, *F*(1, 181) = 7.45, *p =* .007. For sexual identity, participants who identified as plurisexual achieved a same-sex relationship earlier (*M* = 15.28 years, *SE* = 0.32) than participants who identified as monosexual (*M* = 16.23 years, *SE =* 0.36, *p =* .03). Moreover, participants who identified as queer-plus achieved a same-sex relationship earlier (*M* = 14.56 years, *SE* = 0.63) than participants who identified as monosexual (*M* = 16.23 years, *SE =* 0.36, *p =* .02). Last, racial-majority participants achieved this milestone later (*M* = 16.07 years, *SE* = 0.31) than racial-minority participants (*M* = 15.00 years, *SE* = 0.46, *p* = .008).

#### Opposite-sex relationship

The results indicated that there were no significant group differences in age at opposite-sex relationship for sex assigned at birth, *F*(1, 158) = 2.16, *p* = .14. Both school status, *F*(1, 158) = 53.00, *p* < .001, and gender identity, *F*(4, 158) = 3.57, *p* = .008, were found to be significant. However, sexual identity, *F*(3, 158) = 1.05, *p* = .37, and race/ethnicity, *F*(1, 158) = 1.18, *p* = .28, were not significant. Subsequent post hoc analyses yielded no significant differences between groups.

#### Disclosure

The analysis revealed significant group differences in age of disclosure for sex assigned at birth, *F*(1, 351) = 13.40, *p* < .001. Significant group differences in age of disclosure were also found for school status, *F*(1, 351) = 109.99, *p* < .001, gender identity, *F*(4, 351) = 11.40, *p* < .001, and sexual identity, *F*(3, 351) = 3.32, *p* = .02. Race/ethnicity, *F*(1, 351) = 0.74, *p =* .39, was not significant. Post hoc tests indicated that in the case of gender identity, cisgender female participants came out about their sexual identity later (*M* = 15.48 years, *SE* = 0.43) than gender-expansive (*M* = 14.61 years, *SE* = 0.38, *p* = .01) and transmasculine (*M* = 14.07 years, *SE* = 0.47, *p* < .001) participants. For sexual identity, participants who identified as monosexual came out earlier (*M* = 14.39 years, *SE* = 0.26) than participants on the asexual spectrum (*M* = 15.29 years, *SE* = 0.34, *p* = .04).

#### Coming out to an immediate family member

The results indicated significant group differences in age of disclosure to immediate family for sex assigned at birth, *F*(1, 276) = 12.09, *p* < .001, school status, *F*(1, 276) = 111.27, *p* < .001, and gender identity, *F*(4, 276) = 7.63, *p* < .001. Sexual identity, *F*(3, 276) = 0.48, *p =* .70, and race/ethnicity, *F*(1, 276) = 0.0002, *p =* .99, were not significant. Post hoc analyses revealed a significant difference for gender identity, in which transmasculine participants came out about their sexual identity earlier (*M* = 14.83 years, *SE* = 0.47) than cisgender female (*M* = 15.93 years, *SE* = 0.45, *p* = .02) and transfeminine (*M* = 17.44 years, *SE* = 0.74, *p* = .047) participants.

#### Sensitivity analysis

A sensitivity analysis was also run with participants who selected only a bisexual identity (*n* = 58), separate from the plurisexual group, to see whether any discrepancy in findings between plurisexuals and monosexuals could be attributed to how our study categorized sexual identity (for a summary of the statistics, see Section S4). The previously significant finding between participants who identified as plurisexual versus monosexual on age of achievement for the same-sex relationship milestone was not significant when bisexuals were not included. The finding that monosexual participants came out earlier than participants on the asexual spectrum was no longer significant when the bisexual and plurisexual groups were split. Bisexual participants did not significantly differ from monosexual, queer-plus, or plurisexual participants in the timing of any of the SID milestones.

### Sequence of milestone achievement

**Fig. 1.**
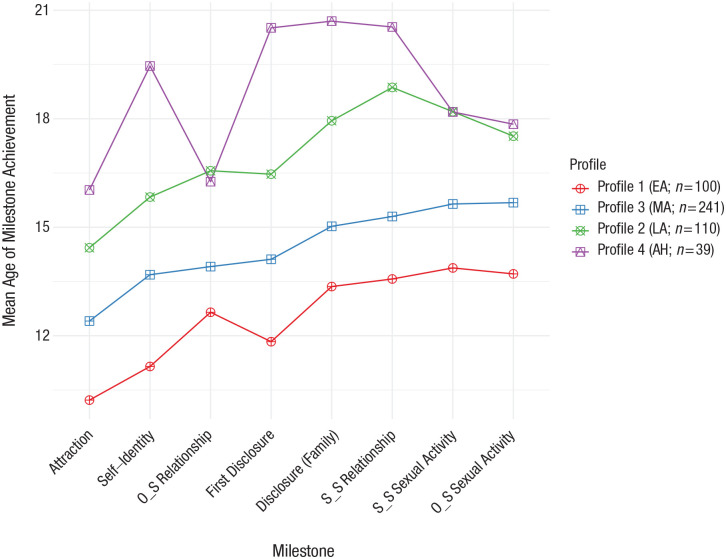
Comparison of mean age of milestone achievement across profiles. Milestones in each profile presented are arranged in order of their achievement. EA = early adolescence; MA = middle adolescence; LA = late adolescence; AH = adulthood; O-S = opposite-sex; S-S = same-sex.

Because of its parsimony and efficiency without significant losses in classification accuracy, lower Bayesian information criterion, and consistent replication, the four-profile model was ultimately chosen (for the model fit indices for the one- to five-profile models, see Table S2). The parameters of each profile are summarized in Table 3, with the SID milestones presented in chronological order for each profile. The demographic characteristics of each profile are presented in Table 4. Consistent with Bishop et al. (2023), the four profiles were best characterized by both the sequencing of the milestones and the ages at which they were achieved: early adolescence (EA; Profile 1), middle adolescence (MA; Profile 3), late adolescence (LA; Profile 2), and adulthood (AH; Profile 4; see Fig. 1). Participants in the EA, MA, and LA profiles reported identity-centered sequencing, in which self-identity occurred before sexual activity. In the AH profile, among participants who reported both milestones, self-identity typically occurred after sexual activity, indicating a sex-centered sequence. However, it is important to highlight that this profile had a high percentage of those not experiencing same-sex sexual activity (with opposite-sex sexual activity also close behind). Therefore, although the AH profile broadly followed a sex-centered sequence, this finding should be interpreted with caution.

**Table 3.** LPA Profile Summaries

Milestone	Age, *M* (*SD*)	Weighted percentage not experiencing milestone, %	Pacing within profile	Difference
Profile 1: EA (*n* = 100; 20.41%)				
Attraction	10.22 (1.43)	7.45	—	—
Self-identity	11.15 (1.16)	0.00	Attraction → self-identity	0.93
First disclosure	11.84 (1.05)	0.98	Self-identity → first disclosure	0.69
O-S Rel	12.65 (2.34)	40.40	First disclosure → O-S Rel	0.81
Familial disclosure	13.36 (1.64)	12.76	O-S Rel → familial	0.71
S-S Rel	13.57 (1.74)	26.29	Familial → S-S Rel	0.21
O-S SA	13.71 (1.96)	58.36	S-S Rel → O-S SA	0.14
S-S SA	13.88 (1.97)	34.04	O-S SA → S-S SA	0.16
Profile 3: MA (*n* = 241; 49.18%)				
Attraction	12.41 (1.67)	9.62	—	—
Self-identity	13.69 (0.99)	0.00	Attraction → self-identity	1.28
O-S Rel	13.91 (2.21)	54.26	Self-identity → O-S Rel	0.22
First disclosure	14.11 (0.95)	0.00	O-S Rel → first disclosure	0.20
Familial disclosure	15.03 (1.33)	15.81	First disclosure → familial	0.91
S-S Rel	15.30 (1.65)	46.49	Familial → S-S Rel	0.27
S-S SA	15.64 (1.77)	48.57	S-S Rel → S-S SA	0.35
O-S SA	15.68 (1.84)	65.85	S-S SA → O-S SA	0.04
Profile 2: LA (*n* = 110; 22.45%)				
Attraction	14.43 (2.22)	11.06	—	—
Self-identity	15.83 (1.18)	0.88	Attraction → self-identity	1.40
First disclosure	16.47 (1.01)	0.00	Self-identity → first disclosure	0.63
O-S Rel	16.56 (2.40)	51.81	First disclosure → O-S Rel	0.09
O-S SA	17.52 (2.73)	48.49	O-S Rel → O-S SA	0.96
Familial disclosure	17.94 (1.97)	32.60	O-S SA → familial	0.43
S-S SA	18.20 (2.25)	45.38	Familial → S-S SA	0.25
S-S Rel	18.87 (2.23)	55.26	S-S SA → S-S Rel	0.67
Profile 4: AH (*n* = 39; 7.96%)				
Attraction	16.03 (2.67)	15.29	—	—
O-S Rel	16.26 (2.61)	40.39	Attraction → O-S Rel	0.23
O-S SA	17.85 (2.87)	46.68	O-S Rel → O-S SA	1.59
S-S SA	18.19 (3.45)	58.18	O-S SA → S-S SA	0.34
Self-identity	19.46 (1.56)	5.12	S-S SA → Self-identity	1.27
First disclosure	20.51 (1.44)	10.09	Self-identity → first disclosure	1.05
S-S Rel	20.54 (2.37)	64.78	First disclosure → S-S Rel	0.02
Familial disclosure	20.70 (2.43)	48.02	S-S Rel → familial	0.16

Note: Milestones in each profile presented are arranged in order of their achievement. LPA = latent profile analysis; EA = early adolescence; O-S Rel = opposite-sex relationship; S-S Rel = same-sex relationship; O-S SA = opposite-sex sexual activity; S-S SA = same-sex sexual activity; MA = middle adolescence; LA = late adolescence; AH = adulthood.

**Table 4.** Demographic Characteristics of Profiles

Demographic	Profile 1: EA	Profile 3: MA	Profile 2: LA	Profile 4: AH
Age in years, *M* (*SD*)	17.39 (1.99)	17.70 (2.39)	20.56 (2.88)	22.67 (2.16)
SAB				
Male	8 (1.63)	43 (8.78)	25 (5.10)	7 (1.43)
Female	90 (18.37)	195 (39.80)	85 (17.35)	32 (6.53)
Intersex	2 (0.41)	—	—	—
Prefer not to say	—	3 (0.61)	—	—
Gender identity				
Cisgender male	7 (1.43)	25 (5.10)	15 (3.06)	3 (0.61)
Cisgender female	24 (4.90)	83 (16.94)	54 (11.02)	21 (4.29)
Gender expansive	39 (7.96)	86 (17.55)	27 (5.51)	10 (2.04)
Transfeminine	—	8 (1.63)	5 (1.02)	2 (0.41)
Transmasculine	29 (5.92)	30 (6.12)	7 (1.43)	2 (0.41)
Uncategorized^a^	1 (0.20)	9 (1.84)	2 (0.41)	1 (0.20)
Sexual identity				
Monosexual	38 (7.76)	80 (16.33)	38 (7.76)	11 (2.24)
Plurisexual	38 (7.76)	110 (22.45)	46 (9.39)	18 (3.67)
Asexual spectrum	16 (3.27)	41 (8.37)	19 (3.88)	4 (0.82)
Queer-plus	8 (1.63)	10 (2.04)	7 (1.43)	6 (1.22)
Race/ethnicity^b^				
White	76 (15.51)	191 (38.98)	91 (18.57)	31 (6.33)
POC	24 (4.90)	47 (9.59)	16 (3.27)	7 (1.43)
Prefer not to say	—	3 (0.61)	3 (0.61)	1 (0.20)
School year				
In high school	69 (14.08)	165 (33.67)	28 (5.71)	2 (0.41)
Not in high school	31 (6.33)	73 (14.90)	81 (16.53)	37 (7.55)
Prefer not to say	—	3 (0.61)	1 (0.20)	—

Note: Values for all demographic characteristics except for age are *n* (%). Percentages presented are for the full sample rather than for individuals within a specific profile. EA = early adolescence; MA = middle adolescence; LA = late adolescence; AH = adulthood; SAB = sex assigned at birth; POC = person of color. ^a^Uncategorized gender identities include those who were intersex at birth and identified their gender as intersex (*n* = 1), did not disclose a gender identity (*n* = 6) or their sex assigned at birth (*n* = 2), or had a lengthy text entry (*n* = 4). ^b^Asian, mixed-race, Black, Indian, and Pasifika/Maori identities and unique text entries were combined as POC because of a low cell count for these groups. A full demographic breakdown of race/ethnicity can be provided on request.

### Profile timing characteristics

The EA profile was characterized by milestone sequencing that began and ended earliest, taking a total of 3.66 years from the first to the last reported milestone. This profile also exhibited the lowest weighted percentage of varied milestone achievement, indicating that, on average, participants in this group reported experiencing a greater number of milestones. The MA profile and LA profile took a total of 3.27 years and 4.43 years, respectively. The AH profile was characterized by milestone sequencing that began and ended latest, taking a total of 4.67 years to progress from the first to the last reported milestone, the longest time frame of all profiles. The AH profile also had the highest weighted percentage of not experiencing a milestone. The LA and AH profiles were distinguished by a higher representation of participants not in high school and who were older on average.

### Variation in milestone experience

**Fig. 2.**
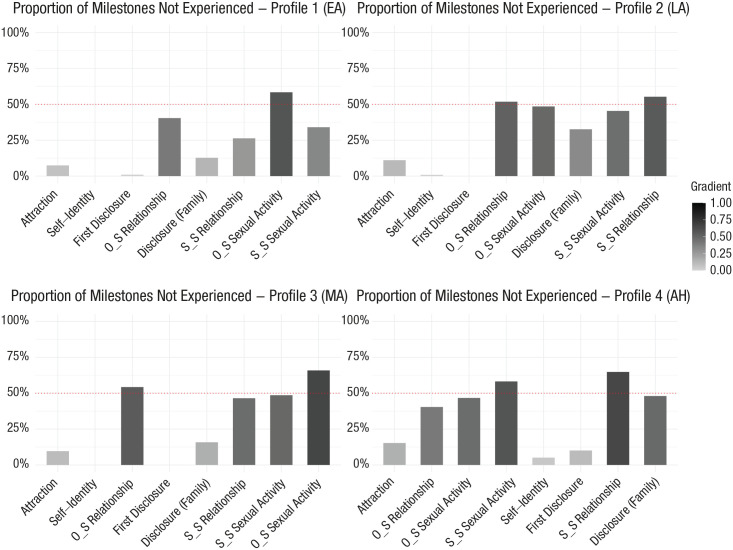
The four latent profiles depicting weighted percentages of milestones not experienced. Milestones in each profile presented are arranged in order of their achievement, as in Figure 1 and Table 3. EA = early adolescence; LA = late adolescence; MA = middle adolescence; AH = adulthood; O-S = opposite-sex; S-S = same-sex.

To further define profiles, they were characterized by the weighted percentage of participants who did not report experiencing specific milestones, with values above 50% indicating milestones not commonly experienced within a given profile. Figure 2 presents the weighted percentages of varied milestone achievement patterns across the four profiles, illustrating the percentage of those who had not experienced a given milestone within each group. These values were adjusted on the basis of each participant’s probability of belonging to their respective profile. The EA profile can be defined by not achieving opposite-sex sexual activity, the MA profile by not achieving an opposite-sex relationship and opposite-sex sexual activity, the LA profile by not achieving an opposite-sex relationship and a same-sex relationship, and the AH profile by not achieving same-sex sexual activity and a same-sex relationship.

### Sensitivity analysis

Last, in regard to the sensitivity analyses, the first attraction milestone was not achieved by 55 participants (11.22% of the sample), and 41 of these identified as being on the asexual spectrum. To explore any potential influence on profile membership, two additional models were run in which the full sample was used with the attraction milestone removed and participants on the asexual spectrum removed, respectively, and all milestones were included (see Table S3 and Figs. S1 and S2). Profiles excluding participants on the asexual spectrum demonstrated similar mean ages for milestone achievement and sequencing compared with the full sample. As expected, the weighted percentages decreased for the attraction milestone across all profiles when excluding participants on the asexual spectrum. The removal of the attraction milestone from the analysis with the full sample led to similar sequencing and mean ages of milestone achievement across all profiles.

### Minority stress outcomes of profile membership

Profile differences were assessed using a series of Wald tests within the LPA framework in Mplus that were specifically designed to evaluate the statistical significance of the discrepancies in outcome measures across different profile memberships. From these analyses (for a summary, see Table 5), rejection sensitivity was found to be higher in the EA profile than in the LA profile and higher in the MA profile than in the LA profile, with other profile comparisons showing no significant differences. Internalized homonegativity did not show significant differences between any profiles. Emotion dysregulation was higher in the EA profile than in the LA profile and in the MA profile. Self-acceptance of sexual identity was higher in the MA profile than in the LA profile and the AH profile. No other comparisons were significant.

**Table 5.** Differences in Outcome Variables Across Profiles

Profile	Rejection sensitivity			Internalized homonegativity			Emotion dysregulation			Sexuality Acceptance		
	Δ	*SE*	*p*	Δ	*SE*	*p*	Δ	*SE*	*p*	Δ	*SE*	*p*
EA vs. LA	**3.12**	**0.96**	.**001**	0.09	0.57	.870	**5.81**	**2.28**	.**011**	2.04	1.43	.154
EA vs. MA	0.89	0.84	.287	0.32	0.53	.552	**3.89**	**1.83**	.**033**	−1.30	1.19	.273
EA vs. AH	1.62	1.43	.257	−0.83	0.77	.280	5.61	3.10	.070	4.02	2.14	.061
LA vs. MA	−**2.23**	**0.77**	.**004**	0.22	0.45	.622	−1.92	2.02	.343	−**3.34**	**1.16**	.**004**
LA vs. AH	−1.50	1.18	.205	−0.93	0.68	.171	−0.21	2.62	.938	1.98	1.92	.302
MA vs. AH	0.73	1.27	.566	−1.15	0.66	.082	1.71	2.86	.549	**5.32**	**1.94**	.**006**

Note: The significance values reported are from the Wald tests done in Mplus. Δ = unstandardized difference in intercepts of a given outcome variable across different latent profiles; *SE* = standard error of the estimated difference in intercepts between profiles for the given outcome variable; EA = early adolescence; MA = middle adolescence; LA = late adolescence; AH = adulthood. Bold entries represent significant findings below *p* < .05.

## Discussion

This study examined common sequences and ages of SID milestone achievements among Generation Z LGBTQ+ participants in Australia, revealing substantial variation in minority stress experiences and SID timing within this cohort. Our findings support the hypothesis that younger Generation Z participants experience SID milestones earlier and more quickly than older counterparts, with EA and MA profiles achieving milestones at younger ages and a faster pace than LA and AH profiles. This trend likely reflects broader societal shifts within Generation Z, such as greater visibility and acceptance of LGBTQ+ identities (Das & Farber, 2020; Riggs & McIntyre, 2022; Twenge et al., 2024). Although these factors may encourage earlier self-identification, they can also expose younger individuals to minority stressors at earlier developmental stages (Russell & Fish, 2019).

Monosexual and plurisexual participants showed similar SID sequences and pacing, with only plurisexuals reaching same-sex relationship milestones slightly earlier than monosexuals. When bisexuals were excluded, no differences in this milestone were observed. Prior studies (e.g., Hall et al., 2021) have found monosexual individuals achieve attraction, self-identity, and sexual activity milestones earlier than bisexuals, although relationship milestones were not previously incorporated. Our findings suggest that earlier same-sex relationship milestones among plurisexuals may reflect a unique integration of social and relational identities, which could foster self-acceptance but also expose individuals to relationship-specific pressures. These dynamics highlight a potential need for tailored support for plurisexual experiences.

Our sensitivity analysis found no significant timing differences in SID milestones among bisexual, monosexual, queer-plus, or plurisexual participants. Although this study examined milestone timing by sexual identity, it did not analyze concurrent differences by gender and sexual identity. Gender identity development research suggests that gender-based analyses within plurisexual groups may reveal distinctive milestone patterns (Layland et al., 2023). Findings by Bishop et al. (2020), largely driven by gay male participants (underrepresented in our sample), suggest that bisexual individuals may share characteristics with other plurisexuals, particularly in same- and opposite-sex relationship contexts (Cipriano et al., 2022; Galupo et al., 2017).

Gender-expansive and transmasculine participants achieved self-identity and first-disclosure milestones approximately 1 year earlier than cisgender females, with transmasculine individuals also disclosing their sexual identity to family members earlier. These patterns may reflect the unique SID trajectories shaped by gender diversity, which can increase visibility and exposure to minority stress in early developmental contexts (Hanna-Walker et al., 2024). The early achievement of these self-identity and disclosure milestones highlights specific challenges for gender-expansive and transmasculine individuals. These findings align with Bishop et al. (2023), who showed that cisgender women and nonbinary/gender-queer participants were more likely to reach milestones in early or middle adolescence compared with cisgender men. This pattern may be linked to experiences of higher peer victimization, broader disclosure, and less concealment of sexual identities among individuals with greater gender nonconformity (Hu et al., 2024; Thoma et al., 2021).

### Profiles of milestone achievement

Four distinct profiles were identified, each of which varied in the average age of SID milestone achievement and in the proportion of milestones not experienced. More than 90% of participants fell into three profiles characterized by milestone achievement within adolescence, highlighting this period’s importance for identity development. These profiles aligned with other studies identifying EA, MA, LA, and AH profiles (Bishop et al., 2020, 2023; Calzo et al., 2011; Grov et al., 2018). Unlike Calzo et al. (2011), who identified delayed disclosure and self-identification among LGB adults, our study showed a shift in Generation Z toward earlier identity disclosure and self-identification, with participants reaching these milestones during adolescence.

Our study’s milestone sequencing and varied achievement patterns differed from previous milestone research. First, the profiles were distinguished by varying frequencies of milestone experiences, including milestones in sexual activity and romantic relationships across same-sex and opposite-sex contexts. These variations across profiles (see Table 4), which persisted despite the consistent distribution of participants who identified as monosexual, plurisexual, and queer-plus and participants who identified as being on the asexual spectrum, suggest that SID pathways do not strictly conform to discrete sexual identity categories. This implies that SID is characterized by a fluid, individualized progression of milestones rather than rigid identity categories. Generation Z’s unique social context thus calls for adaptive frameworks that capture SID diversity within LGBTQ+ identities.

Second, the EA, MA, and LA profiles followed an identity-centered sequence, with self-identity preceding sexual activity, consistent with prior research (Bishop et al., 2023; Savin-Williams, 2019). By contrast, the AH profile followed a sex-centered sequence, with self-identity occurring after sexual activity. The LA profile placed sexual activity mid-sequence, whereas in the AH profile it occurred earlier, before self-identity. However, it is worth noting that in the AH profile more than 50% of participants did not achieve same-sex sexual activity, and more than 50% achieved opposite-sex sexual activity. Although this profile was characterized by an earlier achievement of sexual activity in sequence, this does not necessarily mean all participants in the profile experienced these milestones. In the EA and MA profiles, sexual activity occurred last. The EA and MA profiles also included younger participants (born later), with younger achievement ages, suggesting that sexual activity is even further decentralized as a defining aspect of SID among younger people. However, younger participants who already identify as LGBTQ+ may differ in milestone patterns from those who have yet to recognize or disclose their identity, highlighting a potential limitation in capturing the full diversity of SID trajectories in this sample.

Our sensitivity analysis showed minimal impact on milestone sequencing when asexual individuals and the attraction milestone were excluded, underscoring the need to analyze asexual development independently and acknowledge diversity among this spectrum of individuals (Kelleher et al., 2023). For asexual individuals, the absence of sexual attraction is a significant milestone, distinct from traditional SID sequences (Kelleher & Murphy, 2022). However, our study’s design was limited in fully capturing asexual-specific milestones because the conventional framework we used primarily reflects milestones experienced by those who experience sexual attraction. This limitation reinforces the need for milestone frameworks that better represent diverse sexual identity pathways, including for individuals on the asexual spectrum.

### Minority stress outcomes

Our study highlights that early achievement of SID milestones among Generation Z brings both challenges and benefits. Profiles with earlier milestone ages were associated with higher rejection sensitivity and emotion dysregulation, suggesting increased vulnerability to proximal minority stressors. Early disclosure in Generation Z may perpetuate minority stress levels reported in younger cohorts (Frost et al., 2022; Meyer et al., 2021), exposing them to identity-based discrimination, bullying, and harassment both offline and online (Fisher et al., 2024; McCormick & Barthelemy, 2021). These stressors are particularly salient in high school or home environments where acceptance is not uniform (Abreu et al., 2022; Millers & Lewis, 2025). Consequently, those who disclose early may encounter negative reactions that older cohorts, with more developed coping strategies and support networks, may handle more effectively (Roberts & Christens, 2021). Those achieving milestones later in adolescence typically have more developed emotion regulation abilities (Pozzi et al., 2021), whereas early adolescence is a formative period for developing these skills (Meredith & Silvers, 2024).

Despite these challenges, there are positive aspects to the earlier SID milestones in Generation Z. The MA profile demonstrated higher self-acceptance of sexual identity than profiles with later milestone achievements. Earlier open discourse on identity can facilitate support pathways and community connection (Diamond & Alley, 2022; Reyes et al., 2023), helping to mitigate minority stressors such as internalized homonegativity (Jaspal & Breakwell, 2022). This may explain the lack of significant findings regarding internalized homonegativity across profiles in our study.

### Limitations and future directions

Interpreting conclusions about milestone timing in Generation Z should be done with caution because cross-sectional data limit capturing individuals who may experience milestones later. Future longitudinal studies could better define cohort patterns and clarify causal links between milestone timing and minority stressors. Bishop et al. (2024) emphasized age-matched cohort studies to accurately interpret generational shifts and reduce biases in cross-sectional data. Although our study provides a snapshot of trends, it may underrepresent potential late achievers because of age-related limitations. Additionally, evolving identities, particularly among TGNC individuals, mean that sexual identity may shift over time, especially during gender transitions, during which gender-affirming care can influence attraction and relationship dynamics (Fowler et al., 2024). Longitudinal tracking could better capture how sexual identity shifts alongside gender identity milestones.

The gender distribution in this sample, skewed toward participants assigned female at birth, limits generalizability, especially regarding male identity development. Additionally, cultural factors unique to the Australian context may reduce the applicability of findings to other settings with differing LGBTQ+ visibility, acceptance, and minority stress experiences. The sample used lacked racial diversity. Previous research has shown that racial-majority individuals typically reach milestones later than racial-minority individuals (Bishop et al., 2020; Hall et al., 2021). Therefore, our lack of race/ethnicity findings could be attributed to the sample’s limited diversity and size. It is also essential to note that milestone measures lack standardization across studies, complicating cross-study comparisons. For example, allowing participants to interpret “same-sex” and “opposite-sex” interactions on the basis of personal understanding may introduce variability, particularly among TGNC participants.

## Conclusion

Our study showed that younger Generation Z Australians are experiencing SID milestones earlier and more quickly than older peers, with trends toward earlier sexual identity disclosure. Monosexual and plurisexual individuals did not show distinct SID patterns, and opposite-sex activities were not exclusive to plurisexual identities. Transmasculine and gender-expansive individuals achieved self-identity and disclosure milestones earlier than cisgender females. Four profiles of SID achievement varied in age, sequence, and achievement patterns, indicating SID pathways do not align strictly with traditional sexual identity categories. EA profiles were linked to higher rejection sensitivity and emotion dysregulation, whereas MA showed higher self-acceptance. These findings do not imply causality and are not generalizable to all LGBTQ+ young people or Generation Z. This study lays the groundwork for further research on SID milestones and experiences of proximal minority stressors in Generation Z.

## Supplemental Material

sj-docx-1-pss-10.1177_09567976251372130 – Supplemental material for Sexual Identity Development Milestones, Latent Profiles, and Proximal Minority Stressors in Australia’s Generation Z Supplemental material, sj-docx-1-pss-10.1177_09567976251372130 for Sexual Identity Development Milestones, Latent Profiles, and Proximal Minority Stressors in Australia’s Generation Z by William Warton, Michelle L. Byrne and Kelly-Ann Allen in Psychological Science

## Transparency

*Action Editor:* Ivan Carbajal

*Editor:* Simine Vazire

*Author Contributions*

**William Warton:** Conceptualization; Data curation; Formal analysis; Investigation; Methodology; Project administration; Resources; Validation; Visualization; Writing – original draft; Writing – review & editing.

**Michelle L. Byrne:** Conceptualization; Formal analysis; Funding acquisition; Investigation; Methodology; Project administration; Resources; Supervision; Writing – review & editing.

**Kelly-Ann Allen:** Conceptualization; Investigation; Methodology; Supervision; Writing – review & editing.

**Artificial Intelligence:** No AI-assisted technologies were used in this research or the creation of this article.

**Ethics:** The current study was approved by the Monash University Human Research Ethics Committee (ID 37035).

**Open Practices:** Preregistration: The aims, hypotheses, method, and analyses of this study were registered on July 25, 2023, during data collection but prior to any author viewing the data (https://osf.io/ynjsm). Data collection began on April 29, 2023. Minor deviations from the preregistration in the planned statistical analyses were made to maintain statistical rigor, as detailed in the Method section. Materials: All study materials are publicly available (https://doi.org/10.5281/zenodo.16552435). Data: All primary data are publicly available (https://doi.org/10.5281/zenodo.16552435). Analysis scripts: The analysis scripts used for the primary analyses are publicly available (https://doi.org/10.5281/zenodo.16552435). Computational reproducibility: The computational reproducibility of the results in the main article (but not in the Supplemental Material) has been independently confirmed by the journal’s STAR team.
