# Supplementary material for: Sexual Identity Development Milestones, Latent Profiles, and Proximal Minority Stressors in Australia’s Generation Z
Source: Psychol Sci. 2025 Sep 10;36(10):796–814. doi: 10.1177/09567976251372130 (PMC13428933; doi:10.1177/09567976251372130)
Supplement: Supplementary file 1 [file sj-docx-1-pss-10.1177_09567976251372130.docx]

Supporting Information

**S1: Chi-Square Analysis for Demographic Differences in Milestone Achievement**

Chi-square analyses with a Bonferroni correction testing the differences between participants who did and did not report milestone achievement by demographic characteristics are displayed in main text Table 1. Adjusted p-values are reported below. No differences were found in milestone achievement by sex assigned at birth, religious identity, disability status, and race/ethnicity. Reports of milestone achievement varied by **sexual identity**, including initial attraction (χ²(3, *N* = 488) = 166.29, *p* < .001), first self-identity (χ²(3, *N* = 490) = 18.96, *p* < .001), first same-sex sexual activity (χ²(3, *N* = 488) = 40.52, *p* < .001), first opposite-sex sexual activity (χ²(3, *N* = 484) = 37.13, *p* < .001), and first opposite-sex relationship (χ²(3, *N* = 489) = 30.55, *p* < .001). **Gender identity** differences were found in first same-sex relationship (χ²(4, *N* = 477) = 21.76, *p* < .001) and coming-out to an immediate family member (χ²(4, *N* = 477) = 26.44, *p* < .001).

Differences in milestone achievement for sexual activity were found based on if participants were currently in high school; these were present in both first same-sex sexual activity (χ²(1, *N* = 484) = 37.26, *p* < .001), and first opposite-sex sexual activity (χ²(1, *N* = 481) = 37.96, *p* < .001). Differences were also found in participants based on their neurodivergence (self-identified), including first same-sex relationship (χ²(1, *N* = 437) = 20.76, *p* < .001), and coming-out to an immediate family member (χ²(1, *N* = 437) = 35.54, *p* < .001).

**Table S1.** Demographics Characteristics by Milestone Achievement

|  | **Attraction** | **Self-ID** | **S-S SA** | **O-S SA** | **S-S Rel** | **O-S Rel** | **First Disclosure** | **Familial Disclosure** |  |
| --- | --- | --- | --- | --- | --- | --- | --- | --- | --- |
| **Demographics** | ***n* (%)** | ***n* (%)** | ***n* (%)** | ***n* (%)** | ***n* (%)** | ***n* (%)** | ***n* (%)** | ***n* (%)** | ***N*** |
| **SAB** |  |  |  |  |  |  |  |  |  |
| Male | 76 (15.5) | 82 (16.7) | 46 (9.4) | 30 (6.1) | 39 (8.0) | 33 (6.7) | 81 (16.5) | 57 (11.6) | 83 |
| Female | 355 (72.4) | 400 (81.6) | 203 (41.4) | 151 (30.8) | 209 (42.7) | 192 (39.2) | 396 (80.8) | 314 (64.1) | 402 |
| *X^2^(df), p* | 0.79 (1), = .37 | 0.00 (1), > .99 | 0.61 (1), = .43 | 0.01 (1), = .90 | 0.50 (1), = .48 | 1.51 (1), = .22 | 0.58 (1), = .45 | 2.90 (1), = .09 |  |
| Intersex | 1 (0.2) | 2 (0.4) | 1 (0.2) | - | 1 (0.2) | 2 (0.4) | 2 (0.4) | 2 (0.4) | 2 |
| Prefer not to say | 3 (0.6) | 3 (0.6) | 1 (0.2) | 1 (0.2) | 2 (0.4) | 3 (0.6) | 3 (0.6) | 3 (0.6) | 3 |
| **Gender Identity** |  |  |  |  |  |  |  |  |  |
| Cisgender male | 46 (9.4) | 49 (10.0) | 27 (5.5) | 15 (3.1) | 20 (4.1) | 16 (3.3) | 48 (9.8) | 31 (6.3) | 50 |
| Cisgender female | 165 (33.7) | 180 (36.7) | 85 (17.3) | 69 (14.1) | 76 (15.5) | 84 (17.1) | 179 (36.5) | 126 (25.7) | 182 |
| Gender-Expansive | 137 (28.0) | 162 (33.1) | 85 (17.3) | 60 (12.2) | 93 (19.0) | 82 (16.7) | 160 (32.7) | 136 (27.8) | 162 |
| Transfeminine | 13 (2.7) | 15 (3.1) | 8 (1.6) | 7 (1.4) | 8 (1.6) | 7 (1.4) | 15 (3.1) | 11 (2.2) | 15 |
| Transmasculine | 62 (12.7) | 68 (13.9) | 39 (8.0) | 28 (5.7) | 48 (9.8) | 36 (7.3) | 67 (13.7) | 63 (12.9) | 68 |
| *X^2^(df), p* | 4.12 (4), = .39 | 3.70 (4), = .45 | 2.79 (4), = .59 | 2.65 (4), = .62 | 21.76 (4), **< .001** | 6.51 (4), = .16 | 7.35 (4), = .12 | 26.44 (4), **< .001** |  |
| Uncategorised**^a^** | 12 (2.4) | 13 (2.7) | 7 (1.4) | 3 (0.6) | 6 (1.2) | 5 (1.0) | 13 (2.7) | 9 (1.8) | 13 |
| **Sexual Identity** |  |  |  |  |  |  |  |  |  |
| Monosexual | 164 (33.5) | 167 (34.1) | 103 (21.0) | 46 (9.4) | 102 (20.8) | 56 (11.4) | 165 (33.7) | 133 (27.1) | 167 |
| Plurisexual | 202 (41.2) | 211 (43.1) | 112 (22.9) | 108 (22.0) | 100 (20.4) | 127 (25.9) | 210 (42.9) | 161 (32.9) | 212 |
| Asexual Spectrum | 39 (8.0) | 80 (16.3) | 16 (3.3) | 14 (2.9) | 30 (6.1) | 30 (6.1) | 79 (16.1) | 61 (12.4) | 80 |
| Queer-plus | 30 (6.1) | 29 (5.9) | 20 (4.1) | 14 (2.9) | 19 (3.9) | 17 (3.5) | 28 (5.7) | 21 (4.3) | 31 |
| *X^2^(df), p* | 166.29 (3), **< .001** | 18.96 (3), **< .001** | 40.52 (3), **< .001** | 37.13 (3), **< .001** | 15.17 (3), = .002^c^ | 30.55 (3), **< .001** | 10.89 (3), = .012^c^ | 2.28 (3), = .52 |  |
| **Race/Ethnicity**^b^ |  |  |  |  |  |  |  |  |  |
| White | 347 (70.8) | 388 (79.2) | 204 (41.6) | 145 (29.6) | 202 (41.2) | 181 (36.9) | 382 (78.0) | 311 (63.5) | 389 |
| POC | 82 (16.7) | 93 (19.0) | 46 (9.4) | 35 (7.1) | 48 (9.8) | 47 (9.6) | 94 (19.2) | 63 (12.9) | 94 |
| *X^2^(df), p* | 0.33 (1), = .57 | 0.04 (1), = .84 | 0.29 (1), = .59 | 0.00 (1), > .99 | 0.00 (1), = .97 | 0.22 (1), = .64 | 0.13 (1), = .72 | 6.52 (1), = .011^c^ |  |
| Prefer not to say^e^ | 6 (1.2) | 6 (1.2) | 1 (0.2) | 2 (0.4) | 1 (0.2) | 2 (0.4) | 6 (1.2) | 2 (0.4) | 7 |
| **School Year** |  |  |  |  |  |  |  |  |  |
| In Highschool | 231 (47.1) | 261 (53.3) | 103 (21.0) | 66 (13.5) | 127 (25.9) | 110 (22.4) | 260 (53.1) | 201 (41.0) | 264 |
| Not in Highschool | 201 (41.0) | 222 (45.3) | 148 (30.2) | 116 (23.7) | 123 (25.1) | 118 (24.1) | 218 (44.5) | 173 (35.3) | 222 |
| *X^2^(df), p* | 0.31 (1), = .57 | 1.02 (1), = .31 | 37.26 (1), **< .001** | 37.96 (1), **< .001** | 2.29 (1), = .13 | 6.18 (1), = .013^c^ | 0.00 (1), > .99 | 0.13 (1), = .72 |  |
| Prefer not to say | 3 (0.6) | 4 (0.8) | - | - | 1 (0.2) | 2 (0.4) | 4 (0.8) | 2 (0.4) | 4 |
| **Disability Status** |  |  |  |  |  |  |  |  |  |
| Identified as Disabled | 104 (21.2) | 116 (23.7) | 65 (13.3) | 41 (8.4) | 72 (14.7) | 54 (11.0) | 114 (23.3) | 98 (20.0) | 116 |
| Did not identify | 303 (61.8) | 336 (68.6) | 168 (34.3) | 127 (25.9) | 161 (32.9) | 157 (32.0) | 333 (68.0) | 251 (51.2) | 339 |
| *X^2^(df), p* | 0.00 (1), = 1.00 | 0.12 (1), = .72 | 1.34 (1), = .25 | 0.07 (1), = .80 | 6.78 (1), **=** .009^c^ | 0.00 (1), = 1.00 | 0.62 (1), = .43 | 4.70 (1), = .03^c^ |  |
| Did not disclose | 28 (5.7) | 35 (7.1) | 18 (3.7) | 14 (2.9) | 18 (3.7) | 19 (3.9) | 35 (7.1) | 27 (5.5) | 35 |
| **Neurodivergent** |  |  |  |  |  |  |  |  |  |
| Identified as Neurodivergent | 223 (45.5) | 249 (50.8) | 140 (28.6) | 90 (18.4) | 150 (30.6) | 117 (23.9) | 247 (50.4) | 217 (44.3) | 249 |
| Did not identify | 169 (34.5) | 185 (37.8) | 84 (17.1) | 72 (14.7) | 71 (14.5) | 85 (17.4) | 182 (37.1) | 117 (23.9) | 188 |
| *X^2^(df), p* | 0.00 (1), = 1.00 | 2.00 (1), = .16 | 5.68 (1), = .017^c^ | 0.12 (1), = .73 | 20.76 (1), **< .001** | 0.05 (1), = .83 | 4.54 (1), = .033^c^ | 35.54 (1), **< .001** |  |
| Did not disclose | 43 (8.8) | 53 (10.8) | 27 (5.5) | 20 (4.1) | 30 (6.1) | 28 (5.7) | 53 (10.8) | 42 (8.6) | 53 |
| **Religious Identity** |  |  |  |  |  |  |  |  |  |
| Is religious | 90 (18.4) | 100 (20.4) | 51 (10.4) | 35 (7.1) | 51 (10.4) | 59 (12.0) | 99 (20.2) | 74 (15.1) | 100 |
| Is not religious | 329 (67.1) | 366 (74.7) | 191 (39.0) | 139 (28.4) | 192 (39.2) | 162 (33.1) | 361 (73.7) | 286 (58.4) | 368 |
| *X^2^(df), p* | 0.03 (1), = .86 | 0.00 (1), = 1.00 | 0.01 (1), = .94 | 0.17 (1), = .68 | 0.01 (1), = .92 | 6.38 (1), = .012^c^ | 0.00 (1), = 1.00 | 0.42 (1), = .52 |  |
| Did not disclose | 16 (3.3) | 21 (4.3) | 9 (1.8) | 8 (1.6) | 8 (1.6) | 9 (1.8) | 22 (4.5) | 16 (3.3) | 22 |
| **Total Achieved** | 435 (88.8) | 487^d^ (99.4) | 251 (51.2) | 182 (37.1) | 251 (51.2) | 230 (46.9) | 482 (98.4) | 376 (76.7) |  |

*Note.* SAB = Sex assigned at birth, Self-ID = Self-identity, S-S SA = same-sex sexual activity, O-S SA = opposite-sex sexual activity, S-S Rel = same-sex relationship, O-S Rel = opposite-sex relationship. For each milestone, the number and percentage of participants who achieved the milestone are displayed, with chi-square analysis results (including Bonferroni-corrected p-values) indicating significant associations between demographics and milestone achievement. The rightmost column ("N") offers a summary view of total participants per demographic group.

^a^Uncategorised gender identities include those who were intersex at birth and identified their gender as intersex (*n* = 1), did not disclose a gender identity (*n* = 6), or did not disclose their sex assigned at birth (*n* = 2), or who had a lengthy text entry (*n* = 4). These were not included in the chi-square analysis.

^b^Due to low cell count in the Asian, mixed race, Black, Indian, Pasifika/Maori identities and unique text entries, these were combined into “POC.” A full demographic breakdown of race/ethnicity can be provided upon request.

^c^Not significant after adjusting for multiple comparisons.

^d^Three participants who identified as questioning also did not achieve the self-identity milestone.

^e^Responses that were missing (*n* = 4) and prefer not to say (*n* = 3) were combined.

**S2: Additional Demographics Characteristics for ANCOVA**

***Attraction***

The results indicated significant group differences in age at attraction for sex assigned at birth (*F*(1, 322) = 4.31, *p* = .04), school status (*F*(1, 322) = 35.37, *p* < .001), gender identity (*F*(4, 322) = 4.11, *p* = .003), disability status (*F*(1, 322) = 3.94, *p* = .048), and neurodivergence (*F*(1, 322) = 4.18, *p* = .04). However, no significant effects were found for sexual identity, (*F*(3, 322) = 0.11, *p* = .95), race/ethnicity, (*F*(1, 322) = 0.31, *p* = .58), and religious identity, (*F*(1, 322) = 1.56, *p* = .21). Post-hoc tests indicated that there was a significant difference in age of attraction milestone achievement for participants who self-identified as neurodivergent (*M* = 12.59, *SE* = .32) compared to those who identified as not being neurodivergent (*M* = 13.30, *SE* = .35, *p* = .02).

***Self-Identity***

The results indicated significant group differences in age at self-identification for sex assigned at birth (*F*(1, 357) = 8.59, *p* = .004), school status (*F*(1, 357) = 87.44, *p* < .001), gender identity (*F*(4, 357) = 12.01, *p* < .001), sexual identity (*F*(3, 357) = 3.76, *p* = .01), disability status (*F*(1, 357) = 6.32, *p* = .01), and neurodivergence (*F*(1, 357) = 6.21, *p* = .01). Conversely, no significant effects were found for race/ethnicity, (*F*(1, 357) = 0.45, *p* = .50) and religious identity (*F*(1, 357) = 1.00, *p* = .32). Self-identity was achieved earlier for participants assigned female at birth (*M* = 13.36, *SE* = .35), than those assigned male at birth (*M* = 14.91, *SE* = .47, *p* = .02). Self-identity was achieved later for cisgender-identified female participants (*M* = 15.30, *SE* = .43) than gender-expansive participants (*M* = 14.19, *SE* = .38, *p* < .001), or transmasculine participants (*M* = 13.86, *SE* = .47, *p* < .001). Moreover, participants on the asexual spectrum for their sexual identity achieved self-identity later (*M* = 14.80, *SE* = .34) than those who identified with a monosexual identity (*M* = 13.66, *SE* = .26, *p* = .005). Lastly, participants who self-identified as neurodivergent achieved self-identity earlier (*M* = 13.75, *SE* = .26), than those who did not (*M* = 14.52, *SE* = .28, *p* = .001).

***Same-sex Sexual Activity***

The results indicated no significant group differences in age at same-sex sexual activity for sex assigned at birth (*F*(1, 182) = 2.50, *p* = .12) and gender identity (*F*(4, 182) = 1.77, *p* = .14). Significant effects were observed for school status, (*F*(1, 182) = 30.45, *p* < .001), sexual identity (*F*(3, 182) = 3.51, *p* = .02) and disability status (*F*(1, 182) = 5.40, *p* = .02). Race/ethnicity (*F*(1, 182) = 1.15, *p* = .28), neurodivergence (*F*(1, 182) = 0.91, *p* = .34), and religious identity (*F*(1, 182) = 3.27, *p* = .07), were not significant. Post-hoc tests indicated that there was a significant difference in age of same-sex sexual activity for participants who identified as queer-plus (*M* = 14.57, *SE* = .72), and those who identified with a monosexual identity (*M* = 16.47, *SE* = .44, *p* = .02).

***Opposite-sex Sexual Activity***

The results indicated no significant group differences in age at opposite-sex sexual activity for sex assigned at birth (*F*(1, 129) = 0.82, *p* = .37). Similarly, gender identity, (*F*(4, 129) = 1.82, *p* = .13), sexual identity (*F*(3, 129) = 0.46, *p* = .71), race/ethnicity, (*F*(1, 129) = 0.04, *p* = .85), disability status (*F*(1, 129) = 1.35, *p* = .25), neurodivergence (*F*(1, 129) = 0.80, *p* = .37), and religious identity (*F*(1, 129) = 0.73, *p* = .39), also showed no significant effects. School status (*F*(1, 129) = 30.79, *p* < .001), was found to be significant. However, post-hoc tests revealed that there were no significant differences found between groups.

***Same-sex Relationship***

The results indicated significant group differences in age at same-sex relationship for sex assigned at birth (*F*(1, 181) = 23.59, *p* < .001) and school status, (*F*(1, 181) = 81.70, *p* < .001). Gender identity (*F*(4, 181) = 3.68, *p* = .007), sexual identity (*F*(3, 181) = 3.41, *p* = .02), and race/ethnicity (*F*(1, 181) = 7.45, *p* = .007), also showed a significant effect. Disability status (*F*(1, 181) = 3.53, *p* = .06), neurodivergence (*F*(1, 181) = 1.78, *p* = .18), and religious identity (*F*(1, 181) = 0.23, *p* = .63), were not significant. For sexual identity, participants who identified as plurisexual achieved a same-sex relationship earlier (*M* = 15.28, *SE* = .32) than monosexual participants (*M* = 16.23, *SE* = .36, *p* = .03). Moreover, participants who identified as queer-plus achieved a same-sex relationship earlier (*M* = 14.56, *SE* = .63) than monosexual participants (*M* = 16.23, *SE* = .36, *p* = .02). Lastly, participants who identified as White achieved this milestone later (*M* = 16.07, *SE* = .31), than POC (*M* = 15.00, *SE* = .46, *p* = .008).

***Opposite-sex Relationship***

The results indicated that there were no significant group differences in age at opposite-sex relationship for sex assigned at birth (*F*(1, 158) = 2.16, *p* = .14). Both school status (*F*(1, 158) = 52.99, *p* < .001), and gender identity (*F*(4, 158) = 3.57, *p* = .008), were found to be significant. However, sexual identity (*F*(3, 158) = 1.05, *p* = .37), race/ethnicity (*F*(1, 158) = 1.18, *p* = .28), disability status (*F*(1, 158) = 2.77, *p* = .10), neurodivergence (*F*(1, 158) = 0.15, *p* = .70), and religious identity (*F*(1, 158) = 2.10, *p* = .15), were not significant. Subsequent post-hoc analyses yielded no significant differences between groups.

***Disclosure***

The analysis revealed significant group differences in age of disclosure for sex assigned at birth (*F*(1, 351) = 13.40, *p* < .001). School status (*F*(1, 351) = 109.99, *p* < .001), gender identity (*F*(4, 351) = 11.40, *p* < .001), and sexual identity *F*(3, 351) = 3.32, *p* = .02), also exhibited a significant group difference in age of disclosure. Both race/ethnicity (*F*(1, 351) = 0.74, *p* = .39) and religious identity (*F*(1, 351) = 0.04, *p* = .84), were not significant. Disability status (*F*(1, 351) = 3.77, *p* = .053) and neurodivergence (*F*(1, 351) = 6.14, *p* = .01), were found to be significant. Post-hoc tests indicated that in the case of gender identity, cisgender female participants came out about their sexual identity later (*M* = 15.48, *SE* = .43) than participants who were gender-expansive (*M* = 14.61, *SE* = .38, *p* = .01), and later than participants who were transmasculine (*M* = 14.07, *SE* = .47, *p* < .001). For sexual identity, monosexual participants came out earlier (*M* = 14.39, *SE* = .26) than participants who were on the asexual spectrum (*M* = 15.29, *SE* = .34, *p* = .04). Lastly, participants who identified as neurodivergent came out earlier (*M* = 14.41, *SE* = .26), than those who did not (*M* = 15.19, *SE* = .28, *p* = .001).

***Coming-Out to an Immediate Family Member***

The results indicated significant group differences in age of disclosure to immediate family for sex assigned at birth (*F*(1, 276) = 12.09, *p* < .001), school status (*F*(1, 276) = 111.27, *p* < .001), and gender identity (*F*(4, 276) = 7.63, *p* < .001). Sexual identity, (*F*(3, 276) = 0.48, *p* = .70), race/ethnicity (*F*(1, 276) = 0.0002, *p* = .99), and religious identity, (*F*(1, 276) = 0.02, *p* = .89), were not significant. Additionally, both disability status (*F*(1, 276) = 2.87, *p* = .09), and neurodivergence (*F*(1, 276) = 0.25, *p* = .62) were not significant. Post-hoc analyses revealed a significant difference for gender identity, where transmasculine participants came out about their sexual identity earlier (*M* = 14.83, *SE* = .47), than cisgender female participants (*M* = 15.93, *SE* = .45, *p* = .02).

**S3: Latent Profile Analysis**

Latent Profile Analysis (LPA) belongs to the family of statistical procedures for uncovering hidden (latent) groups called Mixture Models [(Bauer, 2022; Ferguson et al., 2020)](https://www.zotero.org/google-docs/?OKIhbn). In the context of studying SID milestones, Mixture Models utilise a person-centred approach beneficial for examining variations among individuals. This methodology enables the categorisation of individuals based on distinct patterns of their SID milestones, effectively grouping them according to common trajectories or patterns in their milestone achievements. Before conducting LPA, researchers do not know the probabilities associated with each profile, nor the means and (co)variances specific to each profile [(Bauer, 2022; Ferguson et al., 2020)](https://www.zotero.org/google-docs/?CnMX4G). These elements are fundamental parameters that LPA aims to estimate. The model's complexity increases with the number of latent profiles and indicators (observed variables used to determine profile membership). Based on recent recommendations from Bauer (2022), there are three key steps to running an LPA: i) specifying the model, ii) determining the number of latent profiles, and iii) interpreting the target solution substantively.

A critical aspect of LPA is determining the number of latent profiles. For profile enumeration, a decision needs to be made that balances model parsimony and fit while ensuring the identified profiles are meaningfully interpretable. In this case, the most commonly used fit indices are *Bayesian Information Criterion* (BIC) and the *Akaike Information Criterion* (AIC) as these information criteria penalise more complex models. In practice, fit indices can continue to improve as more latent profiles are added (Bauer, 2022). The current consensus in the methodological literature is that model selection should be **equally** guided by interpretability of the profile solution and the fit indices (Bauer, 2022). Therefore, if multiple solutions are found with a similar fit, they should be inspected and a decision made based on substantive grounds (i.e., be theoretically meaningful).

For each milestone, a weighted non-achievement score was calculated for each individual by multiplying the binary non-achievement indicator by the posterior probability of belonging to each profile. To do this the indicators were reversed so that achievement was 0 and non-achievement was 1. The individual weighted scores were then summed across the data for each profile and milestone to get the overall weighted proportion of non-achievement.

**S4: Milestone ANCOVA Data Analysis with Bisexuals Separated from Plurisexuals**

***Attraction***

The results indicated significant group differences in age at attraction for sex assigned at birth (*F*(1, 321) = 4.36, *p* = .038), school status (*F*(1, 321) = 35.76, *p* < .001), and gender identity (*F*(4, 321) = 4.15, *p* = .003). However, no significant effects were found for sexuality group (*F*(4, 321) = 1.40, *p* = .23), race/ethnicity (*F*(1, 321) = 0.43, *p* = .51), or religious identity (*F*(1, 321) = 1.71, *p* = .19). Post-hoc tests indicated that there was a significant difference in age of attraction milestone achievement for participants who self-identified as neurodivergent (*M* = 12.70, *SE* = .31) compared to those who identified as not being neurodivergent (*M* = 13.35, *SE* = .33, *p* = .025).

***Self-Identity***

The results indicated significant group differences in age at self-identity for sex assigned at birth (*F*(1, 356) = 8.58, *p* = .004), school status (*F*(1, 356) = 87.37, *p* < .001), gender identity (*F*(4, 356) = 12.00, *p* < .001), sexual identity (*F*(4, 356) = 3.04, *p* = .018), disability status (*F*(1, 356) = 6.13, *p* = .014), and neurodivergence (*F*(1, 356) = 5.96, *p* = .015). However, no significant effects were observed for race/ethnicity (*F*(1, 356) = 0.40, *p* = .53) or religious identity (*F*(1, 356) = 1.03, *p* = .31). Significant post-hoc comparisons indicated that individuals assigned female at birth self-identified earlier (*M* = 13.41, *SE* = .35) compared to those assigned male at birth (*M* = 14.96, *SE* = .46, *p* = .023). In terms of gender identity, cisgender females self-identified later (*M* = 15.34, *SE* = .43) compared to gender expansive (*M* = 14.25, *SE* = .38, *p* = .001) and transmasculine individuals (*M* = 13.92, *SE* = .47, *p* < .001). Among sexual identities, those on the asexual spectrum self-identified later (*M* = 14.80, *SE* = .34) than monosexuals (*M* = 13.67, *SE* = .26, *p* = .009). Additionally, participants identifying as neurodivergent self-identified earlier (*M* = 13.80, *SE* = .25) compared to those who identified as not being neurodivergent (*M* = 14.56, *SE* = .27, *p* = .002).

***Same-sex Sexual Activity***

The analysis indicated no significant differences in the age of initiating same-sex sexual activity based on sex assigned at birth (*F*(1, 181) = 2.50, *p* = .12) and gender identity (*F*(4, 181) = 1.76, *p* = .14). However, significant effects were found for school status (*F*(1, 181) = 30.36, *p* < .001), sexual identity (*F*(4, 181) = 2.82, *p* = .027), and disability status (*F*(1, 181) = 5.00, *p* = .027). There were no significant effects for race/ethnicity (*F*(1, 181) = 1.22, *p* = .27), neurodivergence (*F*(1, 181) = 0.77, *p* = .38), and religious identity (*F*(1, 181) = 3.14, *p* = .08). Post-hoc tests indicated that there was a significant difference in age of same-sex sexual activity for participants who identified as queer-plus (*M* = 14.55, *SE* = .72), and those who identified with a monosexual identity (*M* = 16.47, *SE* = .44, *p* = .03).

***Opposite-sex Sexual Activity***

The results indicated no significant group differences in age at opposite-sex sexual activity based on sex assigned at birth (*F*(1, 128) = 0.81, *p* = .37). Additionally, no significant differences were found based on gender identity (*F*(4, 128) = 1.81, *p* = .13), sexual identity (*F*(4, 128) = 0.39, *p* = .82), race/ethnicity (*F*(1, 128) = 0.03, *p* = .86), disability status (*F*(1, 128) = 1.48, *p* = .23), neurodivergence (*F*(1, 128) = 0.77, *p* = .38), or religious identity (*F*(1, 128) = 0.78, *p* = .38). However, a significant effect was observed for school status (*F*(1, 128) = 30.60, *p* < .001). Despite this, post-hoc analyses revealed no significant differences in the age of initiating opposite-sex sexual activity between participants still in high school compared to those not in high school.

***Same-sex Relationship***

The results indicated significant group differences in age at same-sex relationship for sex assigned at birth (*F*(1, 180) = 23.48, *p* < .001) and school status, (*F*(1, 180) = 81.32, *p* < .001). Gender identity (*F*(4, 180) = 3.66, *p* = .007), sexual identity (*F*(3, 180) = 2.55, *p* = .041), and race/ethnicity (*F*(1, 180) = 7.41, *p* = .007), also showed significant effects. Disability status (*F*(1, 180) = 3.52, *p* = .062), neurodivergence (*F*(1, 180) = 1.77, *p* = .19), and religious identity (*F*(1, 180) = 0.23, *p* = .63), were not significant. For sexual identity, participants who identified as queer-plus achieved a same-sex relationship earlier (*M* = 14.55, *SE* = .63) than monosexual participants (*M* = 16.22, *SE* = .36, *p* = .039). Participants who identified as White achieved this milestone later (*M* = 16.04, *SE* = .30), than POC (*M* = 14.97, *SE* = .44, *p* = .007).

***Opposite-sex Relationship***

The results indicated that there were no significant group differences in age at opposite-sex relationship for sex assigned at birth (*F*(1, 157) = 2.15, *p* = .14). Both school status (*F*(1, 157) = 52.67, *p* < .001), and gender identity (*F*(4, 157) = 3.55, *p* = .008), were found to be significant. However, sexual identity (*F*(3, 157) = 0.84, *p* = .50), race/ethnicity (*F*(1, 157) = 1.20, *p* = .28), disability status (*F*(1, 157) = 2.90, *p* = .09), neurodivergence (*F*(1, 157) = 0.15, *p* = .70), and religious identity (*F*(1, 157) = 1.95, *p* = .16), were not significant. Subsequent post-hoc analyses yielded no significant differences between groups.

***Disclosure***

The analysis revealed significant group differences in age of disclosure for sex assigned at birth (*F*(1, 350) = 13.41, *p* < .001). School status (*F*(1, 350) = 110.02, *p* < .001), gender identity (*F*(4, 350) = 11.41, *p* < .001), and sexual identity (*F*(3, 350) = 2.75, *p* = .03), also exhibited significant group differences in age of disclosure. Race/ethnicity (*F*(1, 350) = 0.66, *p* = .42), religious identity (*F*(1, 350) = 0.05, *p* = .83), and disability status (*F*(1, 350) = 3.62, *p* = .058) were not significant. Neurodivergence (*F*(1, 350) = 5.82, *p* = .02), was found to be significant. Post-hoc tests indicated that in the case of gender identity, cisgender female participants disclosed their sexual identity later (*M* = 15.54, *SE* = .42) than participants who were gender-expansive (*M* = 14.70, *SE* = .37, *p* = .02), and later than participants who were transmasculine (*M* = 14.16, *SE* = .47, *p* < .001). Lastly, participants who identified as neurodivergent disclosed earlier (*M* = 14.48, *SE* = .25), compared to those who identified as not being neurodivergent (*M* = 15.25, *SE* = .27, *p* = .001).

***Coming-Out to an Immediate Family Member***

The analysis indicated significant group differences in the age of disclosure to immediate family for sex assigned at birth (*F*(1, 275) = 12.05, *p* < .001), school status (*F*(1, 275) = 110.94, *p* < .001), and gender identity (*F*(4, 275) = 7.61, *p* < .001). However, sexual identity (*F*(3, 275) = 0.38, *p* = .82), race/ethnicity (*F*(1, 275) = 0.00, *p* = .98), and religious identity (*F*(1, 275) = 0.02, *p* = .90) were not significant. Additionally, both disability status (*F*(1, 275) = 2.81, *p* = .09) and neurodivergence (*F*(1, 275) = 0.24, *p* = .62) were not significant. Post-hoc analyses revealed a significant difference for gender identity, where transmasculine participants disclosed about their sexual identity to immediate family earlier (*M* = 14.83, *SE* = .47) than cisgender female participants (*M* = 15.92, *SE* = .44, *p* = .019).

**S5: LPA Model Fit Indices**

**Table S2.** LPA Model Fit Indices

| Profile | AIC | BIC | aBIC | Entropy | VLMR | LMR |
| --- | --- | --- | --- | --- | --- | --- |
| 1 | 14093.93 | 14379.15 | 14163.32 | NA | NA | NA |
| 2 | 13144.51 | 13501.04 | 13231.25 | 0.90 | 0.27 | 0.27 |
| 3 | 12885.97 | 13313.80 | 12990.05 | 0.89 | 0.67 | 0.67 |
| **4** | **12582.11** | **13081.24** | **12703.54** | **0.90** | **0.24** | **0.24** |
| 5 | 12548.91 | 13119.35 | 12687.69 | 0.89 | 0.74 | 0.74 |

*Note.* AIC = Akaike Information Criterion; BIC = Bayesian Information Criterion; aBIC = Sample Size Adjusted BIC; VLMR = Vo-Leung-Mendell-Rubin Likelihood Ratio Test; LMR = Lo-Mendell-Rubin Likelihood Ratio Test. Neither model partitioned the data beyond 5% of the total sample per profile.

S6: Sensitivity Analysis Profile Comparisons Across Model Conditions

**Table S3.** LPA Sensitivity Analysis Profile Summaries

|  | **Attraction Removed** | | **Asexual Spectrum Removed** | |
| --- | --- | --- | --- | --- |
| **Milestone** | ***M* (*SD*) / *N* (%)** | **Weighted Non-Exp** | ***M* (*SD*) / *N* (%)** | **Weighted Non-Exp** |
| ***Profile 1*** | 239 (48.78%) |  | 82 (20.00%) |  |
| Attraction | - | - | 10.22 (1.39) | 2.42% |
| Self-ID | 13.75 (0.94) | 0.94% | 11.09 (1.20) | 0.00% |
| First Disclosure | 14.15 (0.90) | 0.52% | 11.74 (1.05) | 1.16% |
| O-S Rel | 13.71 (2.12) | 55.55% | 12.94 (2.35) | 39.34% |
| Familial Disclosure | 15.04 (1.29) | 17.25% | 13.24 (1.61) | 13.55% |
| S-S Rel | 15.32 (1.74) | 46.32% | 13.54 (1.75) | 26.40% |
| O-S SA | 15.58 (1.88) | 66.89% | 14.03 (1.77) | 56.49% |
| S-S SA | 15.61 (1.90) | 48.55% | 14.20 (1.81) | 31.51% |
| ***Profile 2*** | 105 (21.43%) |  | 96 (23.41%) |  |
| Attraction | - | - | 14.35 (2.15) | 0.00% |
| Self-ID | 11.18 (1.21) | 0.00% | 15.77 (1.20) | 0.96% |
| First Disclosure | 11.85 (1.02) | 0.96% | 16.43 (1.08) | 0.00% |
| O-S Rel | 12.84 (2.43) | 38.94% | 16.50 (2.32) | 49.29% |
| O-S SA | 13.95 (2.01) | 57.14% | 17.30 (2.73) | 45.79% |
| Familial Disclosure | 13.35 (1.62) | 12.45% | 17.89 (1.98) | 33.04% |
| S-S SA | 14.03 (1.20) | 34.65% | 18.11 (2.29) | 38.21% |
| S-S Rel | 13.67 (1.80) | 26.19% | 18.64 (2.27) | 49.99% |
| ***Profile 3*** | 110 (22.45%) |  | 198 (48.29%) |  |
| Attraction | - | - | 12.36 (1.68) | 3.43% |
| Self-ID | 15.80 (1.25) | 0.00% | 13.59 (0.98) | 0.00% |
| O-S Rel | 16.60 (2.33) | 48.69% | 13.89 (2.24) | 52.06% |
| First Disclosure | 16.50 (1.00) | 0.83% | 14.08 (0.98) | 0.00% |
| Familial Disclosure | 17.96 (1.90) | 30.94% | 15.03 (1.34) | 14.26% |
| S-S Rel | 18.84 (2.17) | 54.76% | 15.29 (1.72) | 43.86% |
| S-S SA | 18.18 (2.16) | 44.50% | 15.53 (1.85) | 41.76% |
| O-S SA | 17.47 (2.71) | 45.48% | 15.51 (1.97) | 61.08% |
| ***Profile 4*** | 36 (7.35%) |  | 34 (8.29%) |  |
| Attraction | - | - | 16.16 (2.61) | 5.88% |
| O-S Rel | 16.26 (2.61) | 35.86% | 15.75 (2.36) | 40.44% |
| O-S SA | 17.85 (2.87) | 42.57% | 17.74 (2.90) | 43.38% |
| S-S SA | 18.19 (3.45) | 55.20% | 18.07 (3.53) | 54.99% |
| Self-ID | 19.50 (1.56) | 0.00% | 19.50 (1.65) | 5.88% |
| First Disclosure | 20.51 (1.44) | 2.78% | 20.67 (1.47) | 11.60% |
| S-S Rel | 20.53 (2.37) | 63.38% | 20.54 (2.37) | 60.97% |
| Familial Disclosure | 20.70 (2.43) | 44.15% | 20.88 (2.60) | 49.17% |

*Note.* Self-ID = self-identity, S-S SA = same-sex sexual activity, O-S SA = opposite-sex sexual activity, S-S Rel = same-sex relationship, O-S Rel = opposite-sex relationship. Weighted Non-Exp = weighted proportion of not experiencing milestone.

**Figure S1.** Comparison of Mean Age of Milestone Achievement Across Profiles with Attraction Removed

**Figure S2.** Comparison of Mean Age of Milestone Achievement Across Profiles with Asexual Spectrum Participants Removed

**References**

[Bauer, J. (2022). A Primer to Latent Profile and Latent Class Analysis. In M. Goller, E. Kyndt, S. Paloniemi, & C. Damşa (Eds.), *Methods for Researching Professional Learning and Development: Challenges, Applications and Empirical Illustrations* (pp. 243–268). Springer International Publishing. https://doi.org/10.1007/978-3-031-08518-5_11](https://www.zotero.org/google-docs/?vFEaV7)

[Ferguson, S. L., G. Moore, E. W., & Hull, D. M. (2020). Finding latent groups in observed data: A primer on latent profile analysis in Mplus for applied researchers. *International Journal of Behavioral Development*, *44*(5), 458–468. https://doi.org/10.1177/0165025419881721](https://www.zotero.org/google-docs/?vFEaV7)
